# Estimating age-related incidence of HBsAg seroclearance in chronic hepatitis B virus infections of China by using a dynamic compartmental model

**DOI:** 10.1038/s41598-017-03080-6

**Published:** 2017-06-06

**Authors:** Jian Zu, Guihua Zhuang, Peifeng Liang, Fuqiang Cui, Fuzhen Wang, Hui Zheng, Xiaofeng Liang

**Affiliations:** 1 School of Mathematics and Statistics, Xi’an Jiaotong University, Xi’an, Shaanxi 710049 P.R. China; 2 Department of Ecology and Evolution, The University of Chicago, Chicago, IL 60637 USA; 3 School of Public Health, Xi’an Jiaotong University, Health Science Center, Xi’an, Shaanxi 710061 P.R. China; 4 Department of Medical Statistics, Ningxia People’ Hospital, Yinchuan, Ningxia 750002 P.R. China; 5 Chinese Center for Disease Control and Prevention, Beijing, 100050 P.R. China

## Abstract

The age-specific seroclearance pattern of hepatitis B surface antigen (HBsAg) in chronic hepatitis B virus (HBV) infections of China remains unclear. In this study, based on three national serosurvey data of hepatitis B in China, we propose an age- and time-dependent discrete model and use the method of non-linear least squares to estimate the age-specific annual rate of HBsAg seroclearance. We found that the HBsAg seroclearance in chronic HBV infections of China aged 1–59 years occurred at an average annual rate of 1.80% (95% CI, 1.54–2.06%) from 1993 to 2006. The HBsAg seroclearance occurred predominantly in the early childhood, 20–24 and 35–39 year age groups. Moreover, our model estimated that HBsAg seroclearance resulted in 23.38% of the decrease of total HBsAg prevalence for population aged 1–59 years in 2006. It also prevented 9.30% of new HBV infections (about 7.43 million people) and 9.95% of HBV-related deaths (about 0.25 million people) from 1993 to 2006. This study develops a new and efficient method to estimate the age-specific incidence of HBsAg seroclearance at a population-level and evaluate its effect.

## Introduction

Recently, the hepatitis B surface antigen (HBsAg) seroclearance in chronic hepatitis B virus (HBV) infections has attracted increasing interest in both long-term studies of the natural history of HBV infection and in patients receiving antiviral therapy^1–5^. From previous studies, it was found that some chronic HBV carriers might spontaneously clear HBsAg from serum or after antiviral therapy^1, 3–5^. Moreover, it was reported that the annual incidence of HBsAg seroclearance varied from 0.12% to 2.38% in cohorts from Asian countries^2, 5–8^ and from 0.54% to 1.98% in cohorts from Western countries^2, 9, 10^. Particularly, a recent study of 1965 inactive HBsAg carriers from Taiwan showed that the average incidence of HBsAg seroclearance was 1.15% per year^5^. For children under 17 years old, a prospective study of 420 HBV-carrier children from Taiwan indicated that HBsAg seroclearance occurred at an average rate of 0.6% per year^11^. Previous studies revealed that the low HBsAg seroclearance rate might be due to the relatively short period of follow-up^5^. In addition, it was found that the age of infection appeared to be the most significant factor associated with HBsAg seroclearance and the interferon treatment for chronic hepatitis B enhanced HBsAg seroclearance by approximately threefold in Western studies and sixfold in Asian studies^2, 12–14^. In general, accurate estimate of the seroclearance rate of HBsAg would not only help us further understand the natural history of HBV infection and the long-term effect of antiviral therapy, but more important, it would help us assess the burden of hepatitis B and design the prevention and treatment strategies in high endemic areas.

However, in China, there has been little discussion about the age-specific seroclearance pattern of HBsAg in chronic HBV infections^1, 5, 15–19^. Two national serosurveys of hepatitis B in 1992 and 2006 revealed that the prevalence of HBsAg decreased by 11.8% to 26.9% from age 15 to age 59^15, 16^. In addition to the possible effect of hepatitis B vaccination, this obvious decrease might be mainly due to the HBsAg seroclearance during the natural course of infection or after antiviral therapy^1, 2, 5^. Because the HBV-related death rate and natural mortality in the chronic HBV carriers were all estimated to be very low^17, 19–21^. However, the HBsAg seroclearance in chronic HBV infections had long been recognized as a rare event in high endemic areas^5^. A follow-up study of 227 inactive HBsAg carriers from China showed that the HBsAg seroclearance occurred at an average rate of 1.42% per year, but the average follow-up duration was only 6.31 years^18^. According to the study of Chu and Liaw^5^, this study might underestimate the annual rate of HBsAg seroclearance in chronic HBV carriers of China. Moreover, in China little was known about this serological change in children. Therefore, further studies were inquired in order to reexamine the age-specific incidence of HBsAg seroclearance in chronic HBV infections of China.

The objective of this study was to develop a mathematical model to estimate the age-related seroclearance rate of HBsAg in chronic HBV infections of China and evaluate its impact. Particularly, based on the two national survey data of hepatitis B in 1992 and 2006, we used the method of non-linear least squares to estimate this age-specific seroclearance rate.

## Materials and Methods

### Study population and survey data

Our estimation and validation is mainly based on data obtained from three national serosurveys of hepatitis B in China^15, 16^ (see Table 1). The main objective of the third serosurvey was to evaluate the impact of hepatitis B vaccination for infants, from its introduction in 1992 to 2014, therefore, in 2014 only the population aged 1–29 years was investigated. In other two national serosurveys, the population aged 1–59 years was included. The prevalence of HBsAg for newborns can be calculated indirectly, thus the population aged 0 year (0 ≤ ***a*** < 1) were not included in the survey. The participants in the three surveys were local residents living in 160 disease surveillance points in 31 provinces of China, which had been selected by Chinese Center for Disease Control and Prevention (China CDC) to be representative of the general population of China^15, 16^. Particularly, these participants were selected by using the method of multi-stage random sampling. Specifically, in order to understand the prevalence of HBsAg, hepatitis B surface antibody (anti-HBs), and hepatitis B core anti-body (anti-HBc) in the representative population of China, based on the expected HBsAg prevalence for different age groups, firstly, 369 townships were identified from 160 counties by simple random selection; Secondly, one village was randomly selected from each township; Thirdly, families were randomly selected according the sample size for each village; Finally, all family members in selected families were investigated and blood were taken for testing hepatitis B markers^15, 16^. Moreover, in the three surveys the serum specimens were all tested in the National Hepatitis Laboratory of Institute for Viral Disease Control and Prevention, China CDC in Beijing^15, 16^. In 1992, the solid-phase radioimmunoassay (SPRIA) was used to detect HBsAg, anti-HBc and anti-HBs in serum specimens^15^. In 2006 and 2014, we used the method of an enzyme-linked immunosorbent assay (ELISA) to detect HBsAg, anti-HBc and anti-HBs in serum specimens^16^. The demographic data were obtained from the National Bureau of Statistics of China and calibrated based on the national census data of China in 1990, 2000 and 2010^22–26^. The vaccination coverage rate of newborns was obtained from the immunization history report of national serosurvey in 2006^27^. The other data were estimated from published literatures^28–38^.

**Table 1:** The China’s national serosurvey data of hepatitis B in 1992, 2006 and 2014.

Age group (years)	1992				2006			2014		
	No. tested	HBsAg (+) No.	HBsAg (+) %	HBV (+) %	No. tested	HBsAg (+) No.	HBsAg (+) %	No. tested	HBsAg (+) No.	HBsAg (+) %
1–4	3288	318	9.6715	38.47	16376	177	1.0809	12681	48	0.3785
5–9	6398	654	10.2219	45.65	11909	191	1.6038	5443	41	0.7533
10–14	6316	712	11.2730	52.47	11844	399	3.3688	4295	53	1.2340
15–19	4639	480	10.3471	54.93	2942	212	7.2060	2618	51	1.9481
20–24	5691	540	9.4887	58.04	2584	211	8.1656	2820	129	4.5745
25–29	7328	704	9.6070	61.07	4194	346	8.2499	3856	195	5.0571
30–34	6579	700	10.6399	62.85	6215	494	7.9485	—	—	—
35–39	6898	636	9.2201	65.32	6949	573	8.2458	—	—	—
40–49	8680	808	9.3088	67.78	10477	880	8.3994	—	—	—
50–59	5885	446	7.5786	70.69	8285	667	8.0507	—	—	—
Total	61702	5998	9.7209	59.17	81775	4150	5.0749	31713	517	1.6303

Note. HBsAg, hepatitis B surface antigen; HBV, hepatitis B virus; No., number; “—”, not covered in survey.

### Model formulation

**Figure 1:**
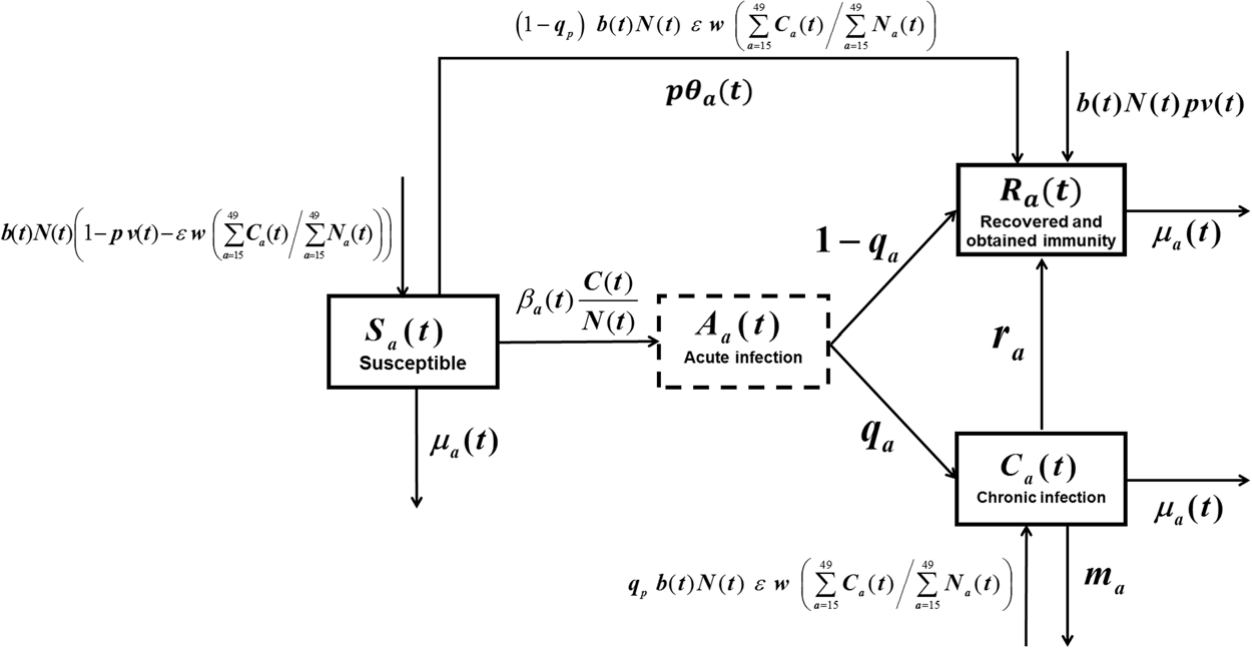
Flow chart of HBV transmission. Here “***a***” denoted the age and “***t***” denoted the time. ***β***_***a***_(***t***) denoted the average transmission rate in age group ***a*** in year ***t*** in the sense that an age ***a*** susceptible had contacted with an infectious individual and was successfully infected; ***q***_***a***_ denoted the age-specific proportion of acute HBV infections that became chronic per year; ***r***_***a***_ denoted the annual rate of HBsAg seroclearance in chronic HBV infections; ***m***_***a***_ denoted the age-specific mortality rate of HBV-related diseases per year; ***θ***_***a***_(***t***) denoted catch-up vaccination coverage rate for population aged 8–15 years in 2009–2011; ***μ***_***a***_(***t***) denoted age- and time-dependent death rate of non-HBV related diseases in year ***t***; ***b***(***t***) denoted birth rate in year ***t***; ***v***(***t***) denoted vaccination coverage rate of newborns in year ***t***; ***p*** denoted vaccination protection rate per year; ***ε*** denoted perinatal infection rate per year; ***w*** denoted the proportion of HBsAg positive mothers in the total prevalence of HBsAg aged 15–49 years; ***q***_***p***_ denoted the proportion of acute HBV infections that became chronic during delivery; ***N***(***t***) denoted the total number of population in year ***t***; ***C***(***t***) denoted the total population number of chronic HBV infections in year ***t***; ***N***_***a***_(***t***) denoted the number of people in age category ***a*** in year ***t***.

According to the natural history of HBV infection and characteristics of HBV transmission in China^1, 2, 15, 16, 20, 21, 27–31, 39–42^, we divided the total population into three compartments: (i) Susceptible to HBV infection ***S***_***a***_(***t***); (ii) Chronic HBV infections ***C***_***a***_(***t***); (iii) Recovered and obtained immunity due to HBV infection or vaccination ***R***_***a***_(***t***), where “***a***” denoted the age and “***t***” denoted the time. In order to directly cite some demographic parameter values^22–26^, here we took “year” as a basic unit of time. Because the average duration of acute HBV infection was about 3 months^1, 19–21, 40^, the acute HBV infection ***A***_***a***_(***t***) was considered as a transient process by which the infected people either recovered and obtained immunity, or became chronic HBV carrier. Particularly, different from our previous work^40^, the perinatal HBV infection and catch-up vaccination for adolescents were considered in this study. Because in 2009–2011 more than 68 million adolescents aged 8–15 years in China received vaccination^28^. Figure 1 illustrated the model structure and parameters. For simplicity of calculation, we assumed that the maximum age of people was 100 years old and divided the population into 101 age groups. The time-step was also set to one year such that if the time increased one year, then the age of people increased one-year-old. Furthermore, the newborns (0 ≤ ***a*** < 1) would enter into one of the three compartments according to whether they were infected during delivery or received vaccination and obtained protection. The susceptible people in age group ***a*** (***a*** = 1, 2, …, 100) might be infected by all age groups of HBV infections, but the susceptible persons of age ***a*** was assumed to be infected at the same transmission rate when they contacted with different age groups of HBV infections. Particularly, we assumed that the people were homogeneously mixed, the contacts for an age ***a*** susceptible (***a*** = 1, 2, …, 100) were divided equally among individuals in the population (i.e. proportional to ***N***_***a***_(***t***)/***N***(***t***) in each age group, where ***N***_***a***_(***t***) was the number of people in age category ***a*** and ***N***(***t***) was the total population), and the probability of a given contact of age ***a*** being infectious was ***C***_***a***_(***t***)/***N***_***a***_(***t***) (where ***C***_***a***_(***t***) was the count of chronic HBV infections at age ***a***), thus a susceptible person of age ***a*** was infected at a rate proportional to ${\boldsymbol{C}}({\boldsymbol{t}})/{\boldsymbol{N}}({\boldsymbol{t}})={\sum }_{{\boldsymbol{a}}=0}^{100}({{\boldsymbol{C}}}_{{\boldsymbol{a}}}({\boldsymbol{t}})/{{\boldsymbol{N}}}_{{\boldsymbol{a}}}({\boldsymbol{t}}))\times ({{\boldsymbol{N}}}_{{\boldsymbol{a}}}\,({\boldsymbol{t}})/{\boldsymbol{N}}({\boldsymbol{t}}))$ (where ***C***(***t***) was the total number of chronic HBV infections**)**. In other words, we used a standard incidence rate to describe this transmission process of HBV^1, 20, 21, 40^. Under the above assumptions, we obtained the following age-structured discrete model of HBV transmission (Eqs (1) and (2)).

For population aged 0 year (0 ≤ ***a*** < 1):

1 $$\{\begin{array}{ccc}{{\boldsymbol{S}}}_{0}({\boldsymbol{t}}+1) & = & {\boldsymbol{b}}({\boldsymbol{t}}){\boldsymbol{N}}({\boldsymbol{t}})({\bf{1}}-{\boldsymbol{p}}{\boldsymbol{v}}({\boldsymbol{t}})-{\boldsymbol{\varepsilon }}w({\sum }_{{\boldsymbol{a}}={\bf{15}}}^{{\bf{49}}}{{\boldsymbol{C}}}_{{\boldsymbol{a}}}({\boldsymbol{t}})/{\sum }_{{\boldsymbol{a}}={\bf{15}}}^{{\bf{49}}}{{\boldsymbol{N}}}_{{\boldsymbol{a}}}({\boldsymbol{t}}))),\\ {{\boldsymbol{C}}}_{0}({\boldsymbol{t}}+1) & = & {{\boldsymbol{q}}}_{{\boldsymbol{p}}}{\boldsymbol{b}}({\boldsymbol{t}}){\boldsymbol{N}}({\boldsymbol{t}}){\boldsymbol{\varepsilon }}w({\sum }_{{\boldsymbol{a}}={\bf{15}}}^{49}{{\boldsymbol{C}}}_{{\boldsymbol{a}}}({\boldsymbol{t}})/{\sum }_{{\boldsymbol{a}}={\bf{15}}}^{49}{{\boldsymbol{N}}}_{{\boldsymbol{a}}}({\boldsymbol{t}})),\\ {{\boldsymbol{R}}}_{0}({\boldsymbol{t}}+1) & = & {\boldsymbol{b}}({\boldsymbol{t}}){\boldsymbol{N}}({\boldsymbol{t}}){\boldsymbol{p}}{\boldsymbol{v}}({\boldsymbol{t}})+({\bf{1}}-{{\boldsymbol{q}}}_{{\boldsymbol{p}}}){\boldsymbol{b}}({\boldsymbol{t}}){\boldsymbol{N}}({\boldsymbol{t}}){\boldsymbol{\varepsilon }}w({\sum }_{{\boldsymbol{a}}={\bf{15}}}^{{\bf{49}}}{{\boldsymbol{C}}}_{{\boldsymbol{a}}}({\boldsymbol{t}})/{\sum }_{{\boldsymbol{a}}={\bf{15}}}^{{\bf{49}}}{{\boldsymbol{N}}}_{{\boldsymbol{a}}}({\boldsymbol{t}})).\end{array}\,$$

For population aged 1−100 years:

2 $$\{\begin{array}{ccc}{{\boldsymbol{S}}}_{{\boldsymbol{a}}+{\bf{1}}}({\boldsymbol{t}}+{\bf{1}}) & = & ({\bf{1}}-{{\boldsymbol{\mu }}}_{{\boldsymbol{a}}}({\boldsymbol{t}})){{\boldsymbol{S}}}_{{\boldsymbol{a}}}({\boldsymbol{t}})-{\boldsymbol{p}}{{\boldsymbol{\theta }}}_{{\boldsymbol{a}}}({\boldsymbol{t}}){{\boldsymbol{S}}}_{{\boldsymbol{a}}}({\boldsymbol{t}})-{{\boldsymbol{\lambda }}}_{{\boldsymbol{a}}}({\boldsymbol{t}}){{\boldsymbol{S}}}_{{\boldsymbol{a}}}({\boldsymbol{t}}),\\ {{\boldsymbol{C}}}_{{\boldsymbol{a}}+{\bf{1}}}({\boldsymbol{t}}+{\bf{1}}) & = & ({\bf{1}}-{{\boldsymbol{\mu }}}_{{\boldsymbol{a}}}({\boldsymbol{t}})){{\boldsymbol{C}}}_{{\boldsymbol{a}}}({\boldsymbol{t}})+{{\boldsymbol{q}}}_{{\boldsymbol{a}}}\,{{\boldsymbol{\lambda }}}_{{\boldsymbol{a}}}({\boldsymbol{t}}){{\boldsymbol{S}}}_{{\boldsymbol{a}}}({\boldsymbol{t}})-{{\boldsymbol{m}}}_{{\boldsymbol{a}}}{{\boldsymbol{C}}}_{{\boldsymbol{a}}}({\boldsymbol{t}})-{{\boldsymbol{r}}}_{{\boldsymbol{a}}}{{\boldsymbol{C}}}_{{\boldsymbol{a}}}({\boldsymbol{t}}),\\ {{\boldsymbol{R}}}_{{\boldsymbol{a}}+{\bf{1}}}({\boldsymbol{t}}+{\bf{1}}) & = & ({\bf{1}}-{{\boldsymbol{\mu }}}_{{\boldsymbol{a}}}({\boldsymbol{t}})){{\boldsymbol{R}}}_{{\boldsymbol{a}}}({\boldsymbol{t}})+{\boldsymbol{p}}{{\boldsymbol{\theta }}}_{{\boldsymbol{a}}}({\boldsymbol{t}}){{\boldsymbol{S}}}_{{\boldsymbol{a}}}({\boldsymbol{t}})+({\bf{1}}-{{\boldsymbol{q}}}_{{\boldsymbol{a}}}){{\boldsymbol{\lambda }}}_{{\boldsymbol{a}}}({\boldsymbol{t}}){{\boldsymbol{S}}}_{{\boldsymbol{a}}}({\boldsymbol{t}})+{{\boldsymbol{r}}}_{{\boldsymbol{a}}}\,{{\boldsymbol{C}}}_{{\boldsymbol{a}}}({\boldsymbol{t}}).\end{array}$$

where ${{\boldsymbol{N}}}_{{\boldsymbol{a}}}({\boldsymbol{t}})={{\boldsymbol{S}}}_{{\boldsymbol{a}}}({\boldsymbol{t}})+{{\boldsymbol{C}}}_{{\boldsymbol{a}}}({\boldsymbol{t}})+{{\boldsymbol{R}}}_{{\boldsymbol{a}}}({\boldsymbol{t}}),{\boldsymbol{N}}({\boldsymbol{t}})={\sum }_{{\boldsymbol{a}}={\bf{0}}}^{{\bf{100}}}{{\boldsymbol{N}}}_{{\boldsymbol{a}}}({\boldsymbol{t}}),{\boldsymbol{C}}({\boldsymbol{t}})={\sum }_{{\boldsymbol{a}}={\bf{0}}}^{{\bf{100}}}{{\boldsymbol{C}}}_{{\boldsymbol{a}}}({\boldsymbol{t}})\,$and ***λ***_***a***_(***t***) = ***β***_***a***_(***t***)***C***(***t***)/***N***(***t***) denoted age- and time-dependent force of HBV infection.

### Initial condition and input parameter estimation

The initial time was taken as ***t***_**0**_ = **1992**. The initial conditions of transmission models (1) and (2) were determined according to the national serosurvey data of hepatitis B in 1992^15^. The age-specific population number in 1992 was calculated according to the national census data of China in 1990^22^ and calibrated according to the fifth and sixth national census data of China in 2000 and 2010^23, 24^. Combining with the prevalence of HBV and HBsAg in 1992, the initial conditions of the model were determined (see Supplementary information 1).

**Figure 2:**
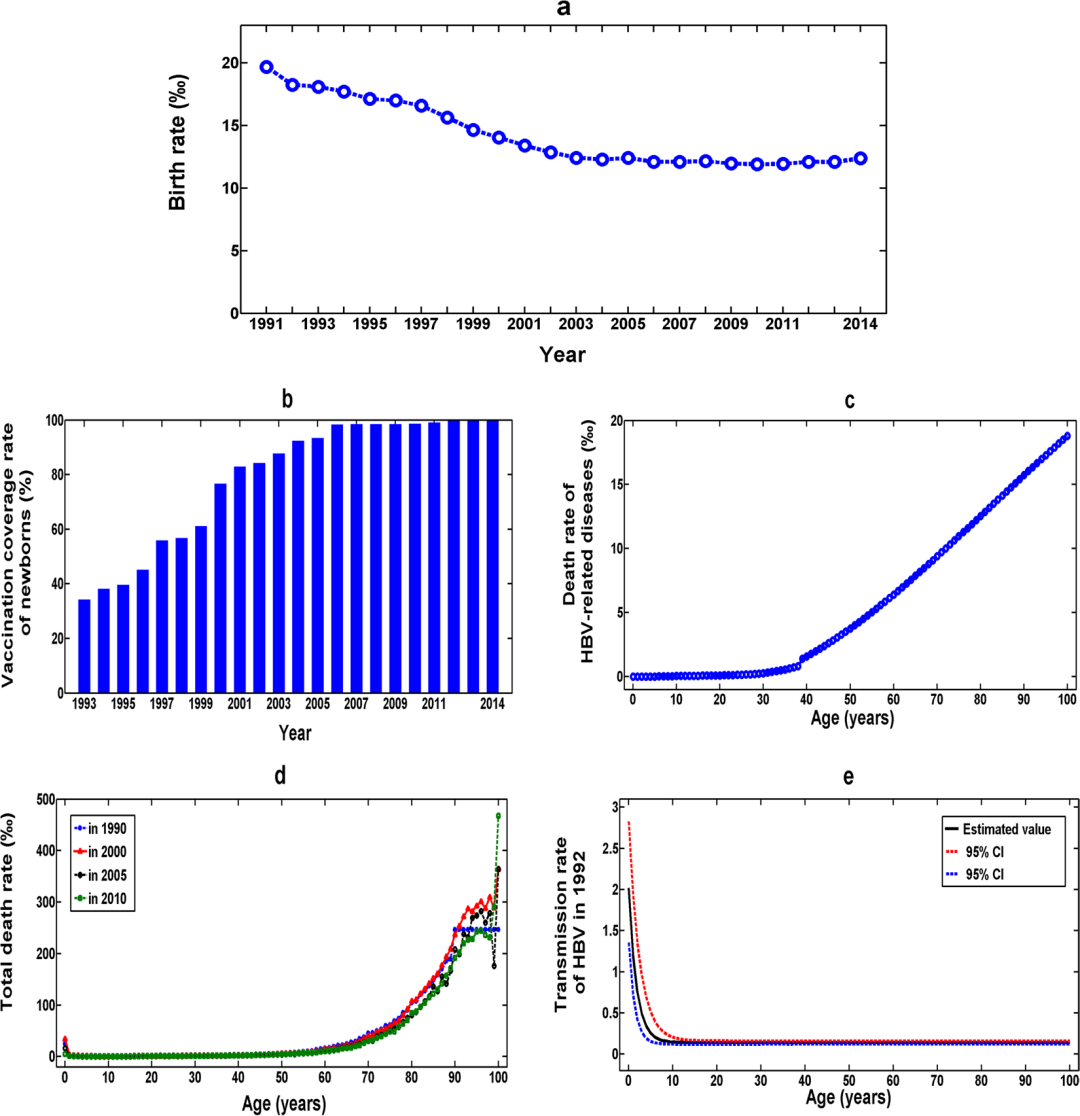
Model input parameters. (**a**) Birth rate from 1991 to 2014. (**b**) Vaccination coverage rate of newborns from 1993 to 2014. (**c**) Age-specific mortality rate of HBV-related diseases. (**d**) The age-specific total death rate in 1990, 2000, 2005 and 2010. (**e**) The age-specific transmission rate of HBV in 1992 and its 95% confidence intervals.

The birth rate per year ***b***(***t***) was obtained from National Bureau of Statistics of China^25^ (see Fig. 2a). The vaccination coverage rate for newborns ***v***(***t***) was collected by a questionnaire and a review of vaccination records^27^ (see Fig. 2b). The age-specific mortality rate of HBV-related diseases per year among chronic HBV infections ***m***_***a***_ was determined from HBV-related cirrhosis and hepatocellular carcinoma mortality curves^21, 29–33^ (see Fig. 2c). The age-specific total death rate per year ***d***_***a***_(***t***) was obtained from the national census data of China in 1990, 2000, 2010 and 1% population sampling survey in 2005^22–26^ (see Table 2 and Fig. 2d). Therefore, the age-specific death rate of non-HBV related diseases was given by ***μ***_***a***_(***t***) = (***d***_***a***_(***t***)***N***_***a***_(***t***) − ***m***_***a***_ ***C***_***a***_(***t***))/***N***_***a***_(***t***).The other parameters except for transmission rate and HBsAg seroclearance rate were determined from published literatures^28–38^. These parameter values were summarized in Table 2.

**Table 2:** Parameter values for input in transmission models (1) and (2) and references.

Description of parameters	Values	References
*b*(*t*): birth rate in year *t*;	See Fig. 2a	25
*v*(*t*): vaccination coverage rate of newborns in year *t*;	See Fig. 2b	27
*p*: vaccination protection rate per year;	0.85 (0.75−0.95)	20, 21, 27, 40
*ε*: perinatal infection rate per year;	0.06 (0.03−0.09)	34–37
*w*: proportion of HBsAg positive mothers in the total prevalence of HBsAg aged 15–49 years;	0.8491 (Calculated according to survey data in 1992)	15
*q*_*p*_:proportion of acute HBV infections that became chronic during delivery period;	0.90	1, 34, 36
*m*_*a*_: age-specific mortality rate of HBV-related diseases per year;	See Fig. 2c	21, 29–33
*d*_*a*_(*t*): age-specific total death rate in year *t*;	See Fig. 2d	22–26
1990 ≤ *t* ≤ 1995	*d*_*a*_(*t*) = *d*_*a*_(1990),	
1996 ≤ *t* ≤ 2002	*d*_*a*_(*t*) = *d*_*a*_(2000),	
2003 ≤ *t* ≤ 2007	*d*_*a*_(*t*) = *d*_*a*_(2005),	
2008 ≤ *t* ≤ 2014	*d*_*a*_(*t*) = *d*_*a*_(2010).	
*μ*_*a*_(*t*): age-specific death rate of non-HBV related diseases in year *t*;	*μ*_*a*_(*t*) = (*d*_*a*_(*t*)*N*_*a*_(*t*) − *m*_*a*_ *C*_*a*_(*t*))/*N*_*a*_(*t*)	21–26, 29–33
*θ*_*a*_(*t*): catch-up vaccination coverage rate for adolescents in year *t*;	0.95 (For population aged 8–15 years in 2009–2011).	28
*q*_*a*_: age-specific proportion of acute HBV infections that became chronic per year;		1, 34, 36
0 ≤ *a* < 1 year	0.30	
1 ≤ *a* ≤ 4 years	0.25	
5 ≤ *a* ≤ 15 years	0.06	
16 ≤ *a* ≤ 100 years	0.03	
*β*_*a*_ (1992): age-specific transmission rate of HBV in 1992.	See Fig. 2e	15, 40

Note. HBsAg, hepatitis B surface antigen; HBV, hepatitis B virus.

In order to estimate the transmission rate of HBV in 1992, ***β***_***a***_(**1992**), notice that the force of HBV infection in 1992, ***λ***_***a***_(**1992**) = ***β***_***a***_(**1992**)***C***(**1992**)/***N***(**1992**), thus we firstly used a catalytic model^40, 43^ to estimate the force of HBV infection in 1992, ***λ***_***a***_(**1992**). This was because if the HBV infection was a natural process and taken to induce a life-long immunity, then the change in the prevalence of HBV infection with age ***a***, ***P***(***a***), with respect to age ***a*** can be described by a catalytic model^43^: ***dP***(***a***)/***da*** = ***λ***(***a***)(1 − ***P***(***a***)), where ***λ***(***a***) is the age-dependent force of HBV infection. The solution of the catalytic model is ${\boldsymbol{P}}({\boldsymbol{a}})={\bf{1}}-\exp (-{\int }_{0}^{{\boldsymbol{a}}}{\boldsymbol{\lambda }}({\boldsymbol{\alpha }})d{\boldsymbol{\alpha }}).$ In addition, the serosurvey in 1992 was performed just before the implementation of routine hepatitis B vaccination for newborns, the survey data characterized the natural HBV infection and was basically in line with the reported result of national serosurvey in 1979^15^. Therefore, the age-specific prevalence of HBV infection in 1992^15^ was used to estimate the force of HBV infection in 1992. Using the same method as that in our previous work (see Section 2. 2 in ref. 40), we obtained the age-specific transmission rate of HBV in 1992 and its 95% confidence intervals, which were depicted in Fig. 2e.

### Estimation of the annual rate of HBsAg seroclearance

In transmission models (1) and (2), the remaining parameters were age-specific seroclearance rate of HBsAg ***r***_***a***_ and transmission rate ***β***_***a***_(***t***). In order to estimate the seroclearance rate of HBsAg, the transmission rate ***β***_***a***_(***t***) were assumed to change in two different ways. Then based on the prevalence of HBsAg aged 1–59 years in 2006 and transmission models (1) and (2), we used the method of non-linear least squares to estimate the average annual rate of HBsAg seroclearance in Chronic HBV infections. The objective function is ${\bf{mi}}{{\bf{n}}}_{{{\boldsymbol{r}}}_{{\boldsymbol{a}}}}{\Vert {{\boldsymbol{P}}}_{{\boldsymbol{s}}}-{{\boldsymbol{P}}}_{{\boldsymbol{m}}}\Vert }_{2}^{2},$ where ***P***_***s***_ was the surveyed prevalence of HBsAg aged 1–59 years in 2006 and ***P***_***m***_ was the estimated prevalence of HBsAg by transmission model (see Supplementary information 2). The annual rate of HBsAg seroclearance in other age groups was assumed to be 1.5%^2^. For simplicity of estimation, according to the grouping approach for national serosurvey of hepatitis B in 1992 and 2006^15, 16^, we divided the population aged 1–59 years into 10 groups, that is, 1–4 years, 5–9 years, 10–14 years, 15–19 years, 20–24 years, 25–29 years, 30–34 years, 35–39 years, 40–49 years and 50–59 years. In each age group we assumed that its seroclearance rate was different. This estimation can be performed with the aid of Matlab function “lsqnonlin”. In this function the maximum number of iterations was taken as 20000 and by default, the algorithm of “trust-region-reflective” was selected (see Supplementary information 2). This algorithm is a subspace trust-region method and is based on the interior-reflective Newton method described in refs 44 and 45. By substituting the estimated parameters, residuals and Jacobian into a Matlab function “nlparci”, we obtained the 95% confidence intervals (CI) of estimated parameters (see Supplementary information 2). All of these calculations and visualization were implemented on the platform of Matlab 2010b (the MathWorks, Inc.). Specifically, we used the following two methods to estimate the annual rate of HBsAg seroclearance.

**Method 1:** We assumed that the transmission rate of HBV remained the same as that in 1992 through the 14-year period, that is, ***β***_***a***_(***t***) = ***β***_***a***_(1992), 1993 ≤ ***t*** ≤ 2006, then based on the prevalence of HBsAg aged 1–59 years in 2006, we estimated the age-specific annual rate of HBsAg seroclearance. We also estimated the total annual rate of HBsAg seroclearance and its 95% confidence interval (CI). In this situation, we might obtain the maximal rate of HBsAg seroclearance.

**Method 2:** We assumed that the transmission rate of HBV decreased exponentially during the 14-year period, that is,

3 $${{\boldsymbol{\beta }}}_{{\boldsymbol{a}}}({\boldsymbol{t}})=({{\boldsymbol{a}}}_{{\bf{1}}}\exp (-{{\boldsymbol{a}}}_{{\bf{2}}}({\boldsymbol{t}}-{\bf{1992}}))+({\bf{1}}-{{\boldsymbol{a}}}_{1})){{\boldsymbol{\beta }}}_{{\boldsymbol{a}}}({\bf{1992}}),\,{\bf{1993}}\le {\boldsymbol{t}}\le {\bf{2006}}{\boldsymbol{.}}$$

This is because in China apart from the implementation of hepatitis B vaccination for newborns, other interventions were also implemented, such as safe injection and HBsAg screening of blood for transfusion, which might reduce the transmission risk of HBV^15, 16, 19–21, 27, 39–42^. Then based on the prevalence of HBsAg aged 1–59 years in 2006, we estimated the age-specific seroclearance rate of HBsAg and parameters ***a***_1_ and ***a***_2_ together. The overall annual rate of HBsAg seroclearance and its 95% CI were also estimated. Particularly, if the lower limit value of confidence interval was negative, then it was set to 0.00. In this situation, we estimated that ***a***_**1**_ = **0.5308** (**95% CI**, **0.3058** − **0.7559**) and ***a***_**2**_ = **0.3853** (**95% CI**, **0.00** − **1.0488**), that is,

4 $${{\boldsymbol{\beta }}}_{{\boldsymbol{a}}}({\boldsymbol{t}})=({\bf{0.5308}}\,\exp (-{\bf{0.3853}}\,({\boldsymbol{t}}-{\bf{1992}}))+{\bf{0.4692}}){{\boldsymbol{\beta }}}_{{\boldsymbol{a}}}({\bf{1992}}),{\bf{1993}}\le {\boldsymbol{t}}\le {\bf{2006}}.$$

Moreover, in order to test the accuracy of our model-based estimation, we compared the estimated HBsAg prevalence by model with national survey data in 2006 and 2014.

### Sensitivity analysis

In order to identify which parameters would affect the estimation of HBsAg seroclearance rate, we performed sensitivity analysis by varying the other parameter values in a reasonable range. Specifically, parameters included the transmission rate, the vaccination coverage rate of newborns, the vaccination protection rate, the perinatal infection rate and the age-specific mortality rate of HBV-related diseases. It should be noted that in sensitivity analysis each parameter was changed, one at a time, while the others were held constant.

### Evaluation of the impact of HBsAg seroclearance

Based on the estimated HBsAg seroclearance rate and transmission rate (4), we used transmission models (1) and (2) to evaluate the impact of HBsAg seroclearance on reducing the HBsAg prevalence and HBV-related deaths. Firstly, through comparison of the HBsAg prevalence for population aged 1–59 years, we estimated how much this prevalence would be reduced due to HBsAg seroclearance and in which age group it would reduce the most in 2006 and 2014. Secondly, we assessed how many people were prevented to be infected by HBV and how many HBV-related deaths were reduced due to HBsAg seroclearance from 1993 to 2014.

## Results

### Estimated annual rate of HBsAg seroclearance

On average, we found that from 1993 to 2006 the HBsAg seroclearance in chronic HBV infections of China aged 1–59 years occurred at an annual rate of 1.80% (95% CI, 1.54–2.06%). The maximal seroclearance rate of HBsAg in chronic HBV infections aged 1–59 years was 2.20% (95% CI, 1.78–2.61%). Moreover, under the assumption that (i) the HBV transmission rate remained the same as that in 1992, and (ii) the HBV transmission rate decreased exponentially, we found that the age-specific seroclearance pattern of HBsAg was similar, except for the 1–4 year age group. The HBsAg seroclearance occurred predominantly in the early childhood, 20–24 and 35–39 year age groups. The estimated annual rates of HBsAg seroclearance in the two scenarios were summarized in Table 3. In some age groups, the rates of HBsAg seroclearance were very low, therefore, their lower limit values of confidence interval might be negative. If the lower limit value of confidence interval was negative, then we set it to 0.00 in Table 3.

**Table 3:** Estimated annual rates of HBsAg seroclearance in chronic HBV infections of China.

Age group (years)	Method 1		Method 2	
	Annual rate (%/yr)	95% CI^*^ (%)	Annual rate (%/yr)	95% CI* (%)
1–4	17.37	(12.00, 22.75)	8.01	(1.44, 14.58)
5–9	3.70	(0.00, 7.70)	4.62	(1.20, 8.04)
10–14	0.00	(0.00, 3.33)	0.56	(0.00, 3.73)
15–19	2.58	(0.00, 6.17)	1.44	(0.00, 4.87)
20–24	3.97	(0.69, 7.26)	4.37	(1.52, 7.21)
25–29	0.00	(0. 00, 2.59)	0.00	(0.00, 2.24)
30–34	0.20	(0. 00, 3.18)	0.00	(0.00, 2.66)
35–39	3.49	(0.58, 6.41)	3.42	(0.90, 5.95)
40–49	0.00	(0.00, 1.26)	0.00	(0.00, 1.07)
50–59	2.72	(1.00, 4.44)	2.58	(1.13, 4.03)
Total	2.20	(1.78, 2.61)	1.80	(1.54, 2.06)

Note. CI, confidence interval; HBsAg, hepatitis B surface antigen; HBV, hepatitis B virus.

^*^If the lower limit value of confidence interval was negative, then it was set to 0.00.

Specifically, from Table 3, we can see that if the transmission rate of HBV remained the same as that in 1992 through the 14-year period (Method 1), then there were three peaks in the age-specific seroclearance rate of HBsAg, the largest peak age distribution was in the 1–4 age group and its annual rate of HBsAg seroclearance was 17.37% (95% CI, 12.00–22.75%), the second and third peak age distributions were respectively in the 20–24 and 35–39 age groups (see Method 1 in Table 3). In this situation, the total annual incidence of HBsAg seroclearance was 2.20% (95% CI, 1.78–2.61%).

If the transmission rate of HBV decreased according to Eq. (4) during the 14-year period (Method 2), we found that the largest peak age distribution was still at 1 to 4 years old and its annual rate of HBsAg seroclearance was 8.01% (95% CI, 1.44–14.58%), the second and third peak age distribution was also in the 20–24 and 35–39 age groups (see Method 2 in Table 3). Compared with the Method 1, it can be seen that if the transmission rate of HBV decreased exponentially since 1993, then the peak age distribution of HBsAg seroclearance rate was the same, but the HBsAg seroclearance rate in the first age group 1–4 years decreased a lot. In this situation, the overall annual rate of HBsAg seroclearance was 1.80% (95% CI, 1.54–2.06%).

**Figure 3:**
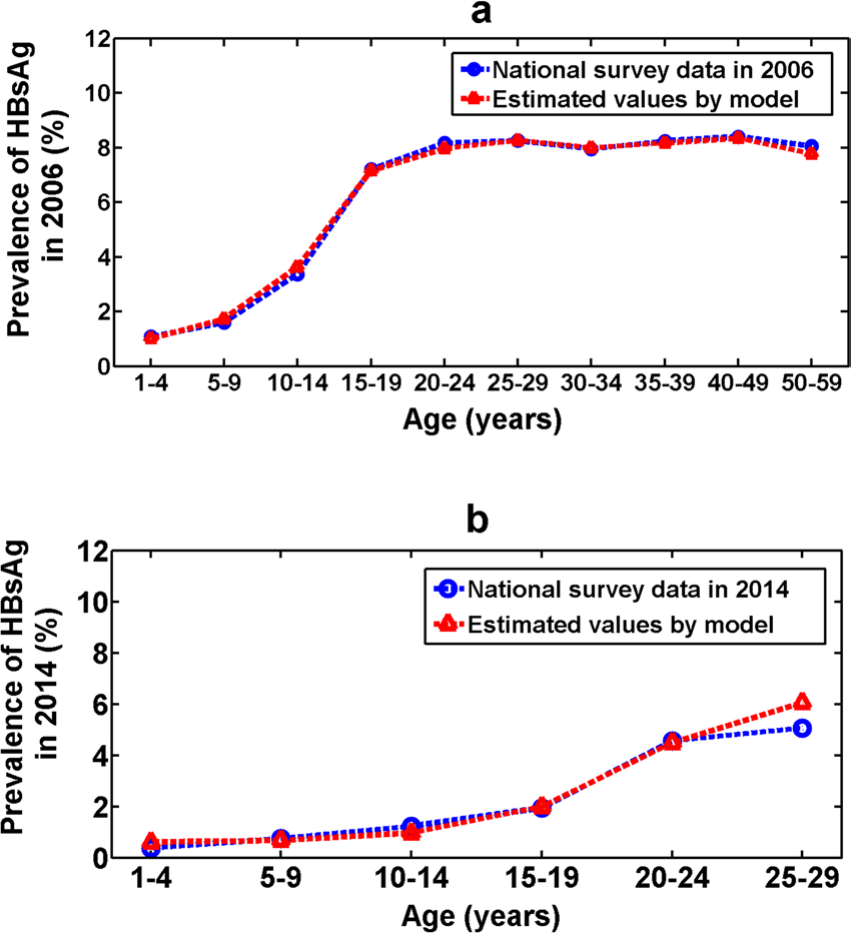
Comparison of estimated HBsAg prevalence with national survey data. (**a**) In 2006. (**b**) In 2014.

By substituting the estimated HBsAg seroclearance rate (Method 2 in Table 3) and transmission rate from Eq. (4) into transmission models (1) and (2), after 14 iterations, we obtained the estimated HBsAg prevalence for population aged 1–59 years in 2006. Compared with the national survey data in 2006, we can see that the estimated values from the model fitted very well with the survey data in 2006 and the maximum absolute error between them was 0.0028, which was in the 50–59 age group (see Fig. 3a). Overall, all the estimated values fell into the 95% confidence intervals of their corresponding survey data in 2006.

Similarly, we compared the estimated HBsAg prevalence for population aged 1 to 29 years with the national survey data in 2014. By substituting the estimated parameter values into transmission models (1) and (2), after 22 iterations, we can see that the estimated HBsAg prevalence for population aged 1 to 29 years was also consistent with the survey data in 2014 (see Fig. 3b). The maximum absolute error between the estimated and surveyed HBsAg prevalence for population aged 1–24 years was 0.0026. The absolute error in the 25–29 age group was 0.0099. The estimated total HBsAg prevalence for population aged 1–29 years was 2.86% in 2014, which fell into the 95% confidence interval of corresponding national survey result in 2014 (95% CI, 2.28–3.06%). This comparison analysis further demonstrated that our model-based estimation with method 2 was credible and it might be used to evaluate the effect of HBsAg seroclearance and predict the prevalence of HBsAg in the future.

### Sensitivity analysis results

Sensitivity analysis indicated that the age-specific transmission rate of HBV was the most sensitive parameter. From Table 3, we can see that under two different assumptions of the HBV transmission rate, the estimated total annual rates of HBsAg seroclearance were respectively 2.20% and 1.80%. Especially, the annual rates of HBsAg seroclearance in the 1–4 age group were respectively 17.37% and 8.01%, which changed the most. For 5–9 and 15–19 age groups there was also some change in their annual rates of HBsAg seroclearance, although the age-specific seroclearance patterns of HBsAg were similar. Therefore, we can say that the age-related transmission pattern of HBV had the most significant influence on the estimation of annual rate of HBsAg seroclearance, especially on the HBsAg seroclearance rate in the early childhood.

**Figure 4:**
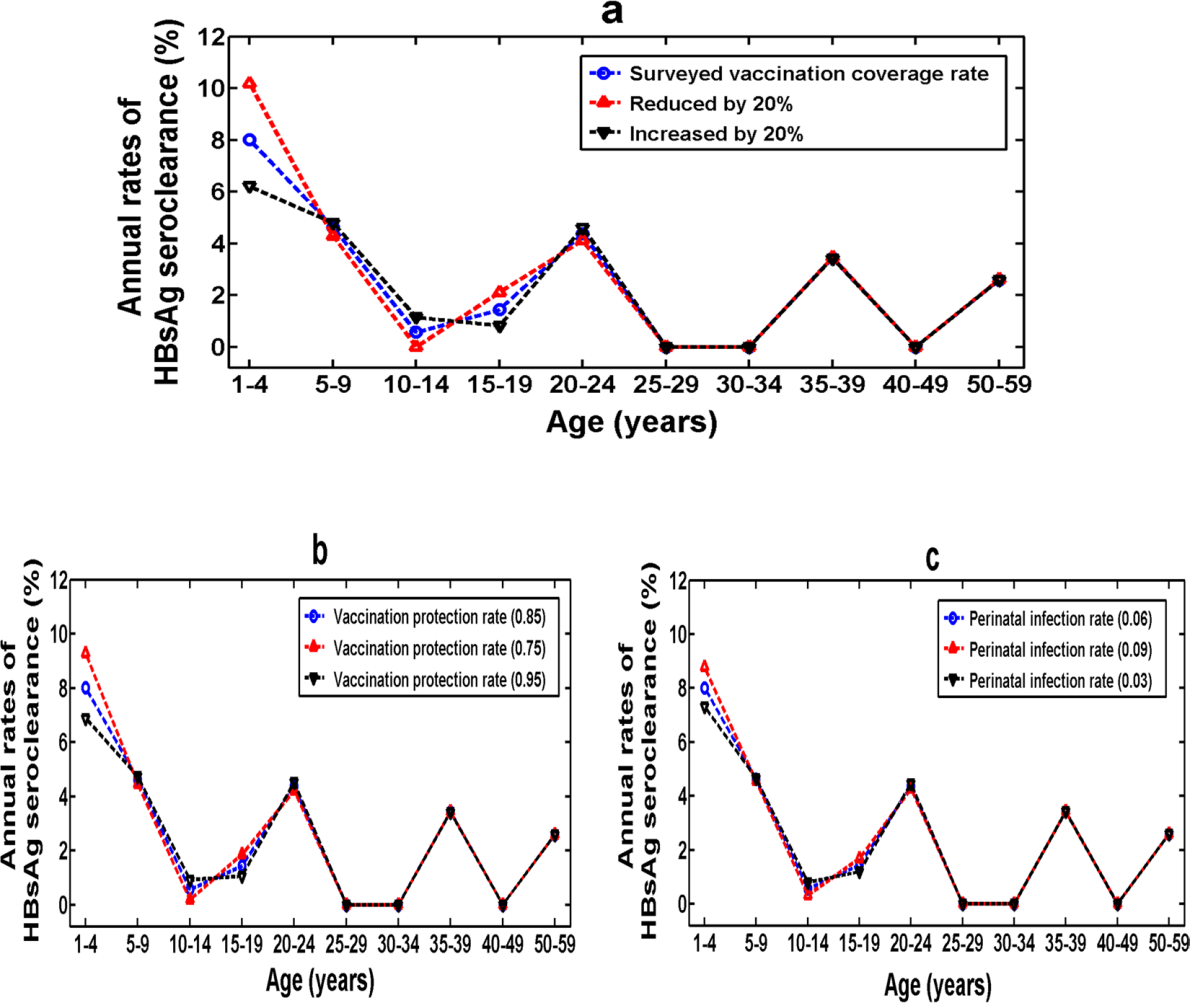
The influence of parameters on estimation of annual rate of HBsAg seroclearance. (**a**) The influence of vaccination coverage rate for newborns. (**b**) The influence of vaccination protection rate. (**c**) The influence of perinatal infection rate.

Moreover, we found that the hepatitis B vaccination coverage rate for newborns and vaccination protection rate had a secondary impact on the estimation of age-specific seroclearance rate of HBsAg. Specifically, if the transmission rate was set to Eq. (4) and the vaccination coverage rate of newborns was cut down by 20% during the 14-year period, then the annual rate of HBsAg seroclearance in the 1–4 age group would increase from 8.01% to 10.18%, which increased by 21.32% (see Fig. 4a). However, if the vaccination coverage rate of newborns was increased by 20% during the 14-year period, then the annual rate of HBsAg seroclearance in the 1–4 age group would decrease from 8.01% to 6.21%, which reduced by 22.47% (see Fig. 4a). In these two cases, the estimated annual rates of HBsAg seroclearance in the 10–14 and 15–19 age groups also changed a bit (see Fig. 4a). Similarly, we can see that if the vaccination protection rate was changed to 0.75 or 0.95, then the estimated annual rate of HBsAg seroclearance in the 1–4 age group changed the most (see Fig. 4b).

We also found that the perinatal infection rate had a certain impact on the estimation of age-specific seroclearance rate of HBsAg. If the perinatal infection rate was changed to 0.03, then the annual rate of HBsAg seroclearance in the 1–4 age group would decrease from 8.01% to 7.32%, which reduced by 8.61% (see Fig. 4c). However, if the perinatal infection rate was changed to 0.09, then the annual rate of HBsAg seroclearance in the 1–4 age group would increase from 8.01% to 8.77%, which increased by 8.67% (see Fig. 4c). In addition, if the perinatal infection rate was changed to 0. 03 or 0.09, the estimated annual rates of HBsAg seroclearance in the 10–14 and 15–19 age groups also changed a bit (see Fig. 4c).

However, if the age-specific mortality rate of HBV-related diseases was increased or reduced by 20%, we found that the age-specific mortality rate of HBV-related diseases had almost no influence on the estimation result of HBsAg seroclearance rate.

### Effect of HBsAg seroclearance

**Figure 5:**
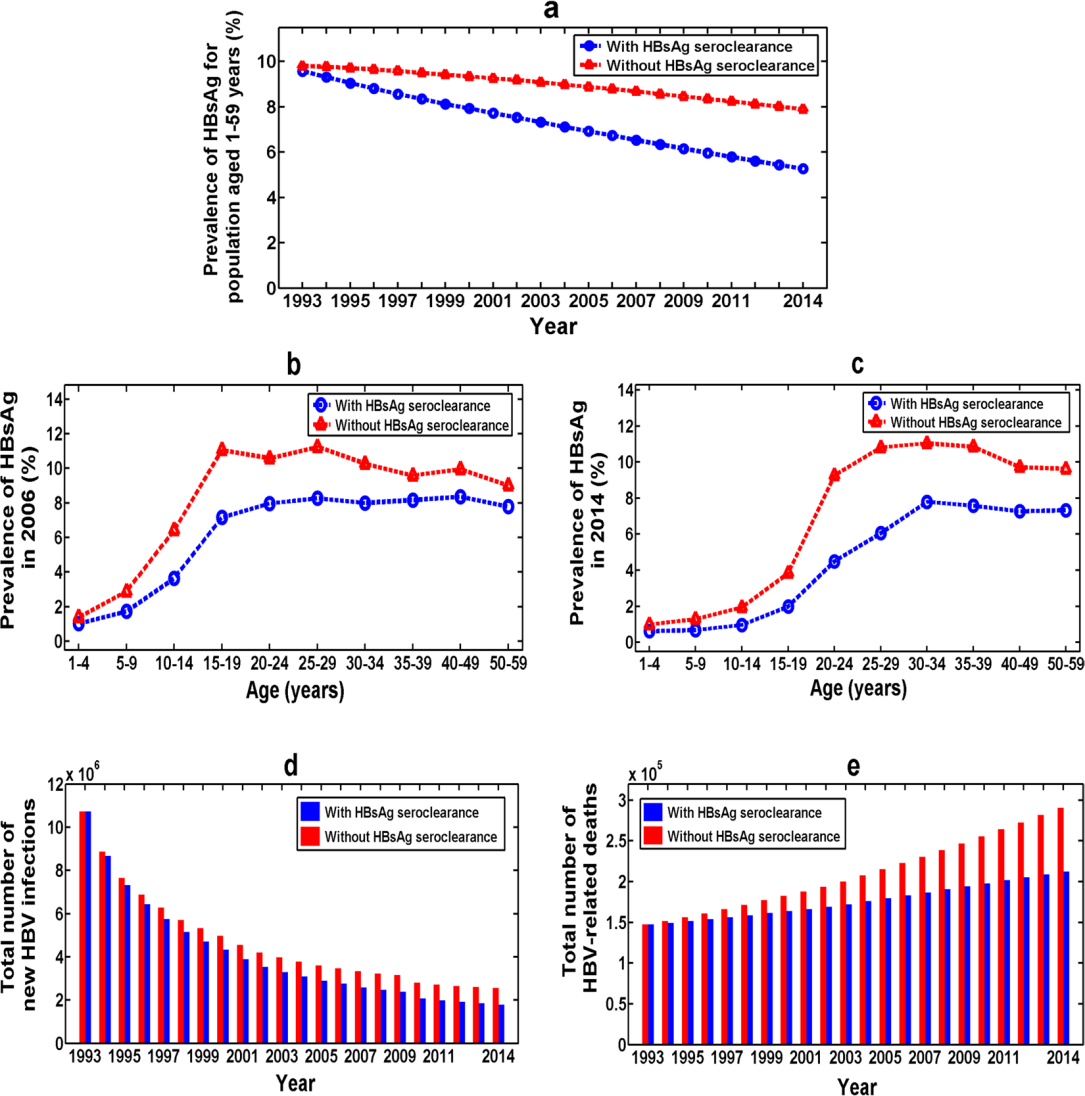
The effect of HBsAg seroclearance. (**a**) On total prevalence of HBsAg for population aged 1–59 years in China. (**b**) On the age-specific prevalence of HBsAg in 2006. (**c**) On the age-specific prevalence of HBsAg in 2014. (**d**) On the total number of new HBV infections. (**e**) On the total number of HBV-related deaths.

Firstly, based on our estimated HBsAg seroclearance rate (Method 2 in Table 3) and transmission rate from Eq. (4), we found that without HBsAg seroclearance in 2006 the total prevalence of HBsAg for population aged 1–59 years in China was 8.78% and in 2014 it would be 7.89% (see Fig. 5a). HBsAg seroclearance resulted in around 23.35% of the reduction of overall HBsAg prevalence in 2006 (8.78% vs 6.73%), and about 33.21% of that in 2014 (7.89% vs 5.27%). This implies that under the current HBsAg seroclearance rate the longer the time, the greater the impact of HBsAg seroclearance on reducing HBsAg prevalence.

Secondly, we found that without HBsAg seroclearance in 2006 the prevalence of HBsAg in the 5–9, 10–14 and 15–19 age groups was respectively 2.88%, 6.41% and 11.06% (see Fig. 5b). HBsAg seroclearance led to respectively 39.96%, 43.29% and 35.24% of the reduction of their HBsAg prevalence in 2006 (see Fig. 5b). In particular, from the point view of an absolute quantity we found that the prevalence of HBsAg in the 15–19 age group reduced the most in 2006 (11.06% vs 7.16%) (see Fig. 5b). Similarly, we found that in 2014 the prevalence of HBsAg in the 20–24 age group reduced the most due to the HBsAg seroclearance (9.26% vs 4.49%) (see Fig. 5c).

Thirdly, we found that if there was no HBsAg seroclearance, our model estimated that 79.92 million people would become HBV-infected from 1993 to 2006 and 102.96 million people would become HBV-infected from 1993 to 2014 (see Fig. 5d). That is to say, HBsAg seroclearance prevented about 9.30% of new HBV infections (7.43 million people) from 1993 to 2006 and around 13.00% of new HBV infections (13.4 million people) from 1993 to 2014 (see Fig. 5d).

Finally, we found that if there was no HBsAg seroclearance, our model estimated that 2.54 million people would die from HBV-related diseases from 1993 to 2006 and 4.62 million people would die from HBV-related diseases from 1993 to 2014 (see Fig. 5e). In other words, HBsAg seroclearance prevented about 9.95% of HBV-related deaths (0.25 million people) from 1993 to 2006 and about 15.90% of HBV-related deaths (0.74 million people) from 1993 to 2014 (see Fig. 5e). More interestingly, from Fig. 5e we can see that under the current rate of HBsAg seroclearance, the longer the time, the more the HBV-related deaths would reduce.

By the above analysis, we can see that the HBsAg seroclearance played an important role in reducing the HBsAg prevalence and HBV-related deaths.

## Discussion

Understanding the general rule of HBsAg seroclearance in chronic HBV infections of China is crucial for the prevention and treatment of hepatitis B. This study developed a dynamic compartmental model to estimate the age-specific annual rate of HBsAg seroclearance in chronic HBV infections of China, which was different from the long-term follow-up study. Firstly, based on the national survey data of hepatitis B in 1992 and 2006, we used the method of non-linear least squares to estimate the annual rate of HBsAg seroclearance in chronic HBV infections aged 1 to 59 years. Then we used the national survey data of hepatitis B in 2014 to test the accuracy of our model-based estimation. This modeling-based study provided countries with a new and practical method to estimate the age-specific HBsAg seroclearance rate, which is more efficient and easier than the long-term follow-up study^5, 18^.

This study showed that from 1993 to 2006 the annual rate of HBsAg seroclearance inchronic HBV carriers of China was appreciably high. Under the assumption that (i) the HBV transmission rate remained the same as that in 1992, and (ii) the HBV transmission rate decreased exponentially, we found that the age-specific seroclearance patterns of HBsAg were similar, except for the 1–4 year age group. Through comparison analysis of the two methods, we can see that the HBsAg seroclearance occurred predominantly in the early childhood, 20–24 and 35–39 year age groups. This analysis also revealed that the age-specific transmission rate of HBV had a certain influence on the estimation of the annual rate of HBsAg seroclearance. Further sensitivity analysis revealed that the vaccination coverage rate of newborns, the vaccination protection rate and perinatal infection rate might also have a certain influence on the estimation of the annual rate of HBsAg seroclearance, especially on the HBsAg seroclearance rate in the early childhood. This might be due to the high incidence of HBV infection in the early childhood. However, the underlying mechanisms that led to a high rate of HBsAg seroclearance in the early childhood warranted further investigation. Particularly, a further long-term follow-up study especially in the children would help to further validate the results of this study.

Most interestingly, if the transmission rate of HBV was assumed to decrease exponentially, by substituting the estimated annual rate of HBsAg seroclearance and transmission rate from Eq. (4) into transmission models (1) and (2), we found that the estimated and surveyed prevalence of HBsAg agreed well with each other for both 2006 and 2014. Therefore, our model-based estimation with method 2 was credible and it might be used to evaluate the effect of HBsAg seroclearance and predict the HBsAg prevalence of China in the future^19–21, 39^.

Moreover, we found that the HBsAg seroclearance played an important role in reducing the HBsAg prevalence and HBV-related deaths. In particular, we found that under the current rate of HBsAg seroclearance, the longer the time, the more the HBV-related deaths would reduce. Previous studies indicated that the conventional interferon treatment for chronic hepatitis B had been shown to enhance HBsAg seroclearance by approximately threefold in Western Countries and sixfold in Asian countries^2, 12–14^. However, in China from 1993 to 2006 less than 10.0% of chronic HBV-infected patients received antiviral therapy due to the high cost of treatment^15, 16, 19–21, 39, 40^. Therefore, we believed that if we increased the treatment coverage rate for chronic HBV-infected patients, then the HBsAg seroclearance rate would gradually increase. If more and more chronic HBV-infected patients were tested and treated earlier, then it would prevent more people to die from HBV-related diseases^19–21, 27, 39–42^.

There were some limitations to be noted. First, this study only estimated the annual rate of HBsAg seroclearance for population aged 1 to 59 years. This was because the maximum age in the national serosurvey of Hepatitis B was 59 years old^16^. If we obtained the prevalence of HBsAg for population aged >59 years, then we can use the same method as in this study to estimate the annual rate of HBsAg seroclearance for population aged >59 years. Second, we did not consider the acute HBV infection as a compartment of the model. Because the average duration of acute HBV infection is 3 months^1, 19–21, 39–41^, the compartment of acute HBV infection was considered as a transient process. In addition, it was almost impossible to determine the initial number of people staying in this state from the national survey data in 1992^15^. Third, for simplicity of estimation, we assumed that the transmission rate decreased in all age groups at the same rate. More realistically, the transmission rate in different age groups might change differently. Fourth, in this study we assumed that the susceptible population aged 1–100 years might be infected by all of the HBV infections, but we did not consider the age-to-age specific transmission rate. However, in the average sense this assumption would not significantly influence our estimation of the HBsAg seroclearance rate. Fifth, in 1992 and 2006 we used two different methods to test serum specimens, the change in testing sensitivity might influence the surveyed results and thus influenced the estimated results. Another limitation of this study was that we did not perform uncertainty analysis in parameters.

In conclusion, this study provided a novel and efficient method to estimate the age-specific annual rate of HBsAg seroclearance at a population-level, which helped us further understand the general pattern of HBsAg seroclearance in the general population of China. Our modeling-based study revealed that the annual rate of HBsAg seroclearance in chronic HBV infections of China was appreciably high. The HBsAg seroclearance played an important role in reducing the HBsAg prevalence and HBV-related deaths. Particularly, this study demonstrated that the HBsAg seroclearance occurred predominantly in the early childhood, 20–24 and 35–39 year age groups. These findings provided some quantitative and new information that might be useful for improving the prevention and treatment strategies of hepatitis B in China and other high endemic areas.

## Electronic supplementary material

Supplementary information
