# Supplementary material for: Estimating age-related incidence of HBsAg seroclearance in chronic hepatitis B virus infections of China by using a dynamic compartmental model
Source: Sci Rep. 2017 Jun 6;7:2912. doi: 10.1038/s41598-017-03080-6 (PMC5460177; doi:10.1038/s41598-017-03080-6)
Supplement: Supplementary file 1 — Supplementary information [file 41598_2017_3080_MOESM1_ESM.pdf]

Estimating age-related incidence of HBsAg seroclearance in chronic hepatitis B virus infections of China by using a dynamic compartmental model

Jian Zu[1, 2, *], Guihua Zhuang[3, *], Peifeng Liang[4], Fuqiang Cui[5], Fuzhen Wang[5], Hui Zheng[5], Xiaofeng Liang[5, *]

Supplementary information 1: Estimation of initial conditions of models (1) and (2)

Firstly, the total number of population in 1992 was calculated based on the national census data of China in 1990 and calibrated according to the national census data of China in 2000 and 2010 (see Supplemental Fig. 1a). Secondly, the initial number of chronic HBV infections equaled to the age-specific prevalence rate of HBsAg in 1992 multiplied by the corresponding population number in 1992. The initial number of recovered population equaled to the difference between the prevalence of HBV and HBsAg multiplied by the corresponding population number in 1992. The prevalence of HBV and HBsAg in 1992 were described in Table 1 of main text. Because no people aged >59 years were enrolled in the serosurvey in 1992, the HBV test data of people aged 50–59 years were used in these old people aged >59 years. The remaining population was the initial susceptible population (see Supplemental Fig. 1b).

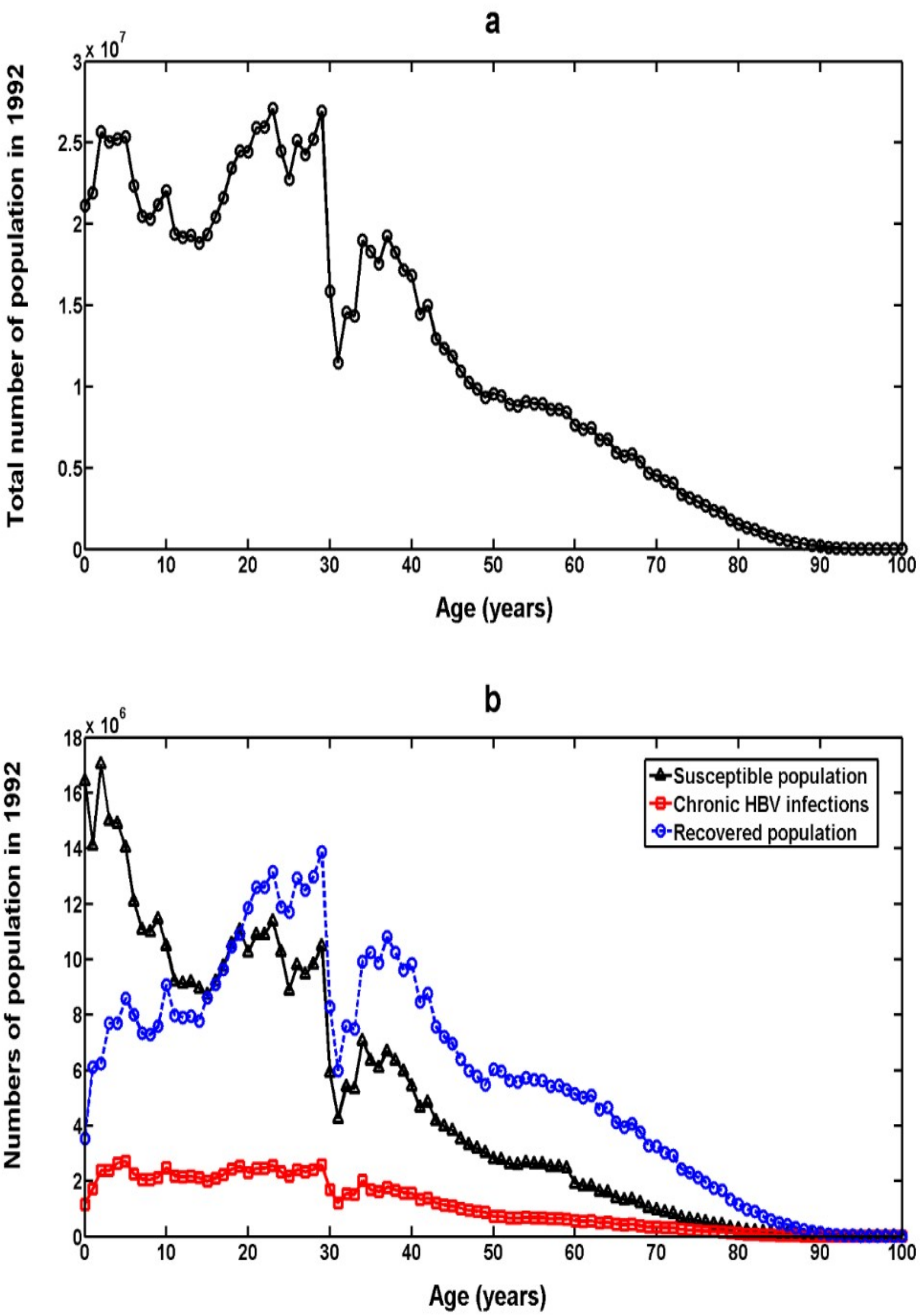

Supplemental Figure 1. Estimated initial conditions of models (1) and (2). (**a**) The total number of population in 1992. (**b**) Initial population numbers of models (1) and (2).

Supplementary information 2: Matlab code for estimation of age-specific seroclearance rate of HBsAg

```
% Main function
function HBVSCRClearancerateLSQNONLIN
clear all
clc
lb=[0 0 0 0 0 0 0 0 0 0 0 0]; % Lower bounds of clearance rate r_a and pamameters a_1 and a_2
ub=[1 1 1 1 1 1 1 1 1 1 1 1]; % Upper bounds of clearance rate r_a and pamameters a_1 and a_2
paras0=[0.015 0.015 0.015 0.015 0.015 0.015 0.015 0.015 0.015 0.015 0.2 0.1]; % Initial point for clearance rate r_a and pamameters a_1 and a_2
options=optimset('Algorithm','trust-region-reflective','Display','final','MaxIter',20000,'MaxFunEvals',20000); % Optimization options
[PARAS,RESNORM,RESIDUAL,EXITFLAG,OUTPUT,LAMBDA,JACOBIAN]=lsqnonlin(@HBVSCRClearancerates,paras0,lb,ub,options); % Solve nonlinear least-squares problem
Estimated_Paras=PARAS % Estimated age-specific seroclearance rates of HBsAg
CI=nlparci(PARAS,RESIDUAL,JACOBIAN) % Confidence intervals of estimated parameters
end

% Objective function
function Diff=HBVSCRClearancerates(PARAS,Ps)
N=14; % From 1993 to 2006
maxage=101;
load HBVdata20171.mat  % Input parameter values and initial conditions
load HBVdata20172.mat  % Age-specific total death rate
ra1=PARAS(1);ra2=PARAS(2);ra3=PARAS(3);ra4=PARAS(4);ra5=PARAS(5);ra6=PARAS(6);ra7=PARAS(7);ra8=PARAS(8);ra9=PARAS(9);ra10=PARAS(10);a1=PARAS(11);a2=PARAS(12);
birthrate=HBVdata20171(1:22,1); % Birth rate
vaccrate=0.85*HBVdata20171(1:22,2); % Vaccination and protection rate of newborns
pirpy=0.06; % Perinatal infection rate per year
hbvmr=HBVdata20171(:,3); % Age-specific mortality rate of HBV-related diseases
acap=HBVdata20171(:,4); % Age-specific proportion of acute HBV infections that became chronic
ra11=0.015; % Clearance rate of HBsAg in other age groups
clearancerate=[ra11,ra1,ra1,ra1,ra1,ra2,ra2,ra2,ra2,ra2,ra3,ra3,ra3,ra3,ra3,ra4,ra4,ra4,ra4,ra4,ra5,ra5,ra5,ra5,ra5,ra6,ra6,ra6,ra6,ra6,ra7,ra7,ra7,ra7,ra7,ra8,ra8,ra8,ra8,ra8,ra9,ra9,ra9,ra9,ra9,ra9,ra9,ra9,ra9,ra10,ra10,ra10,ra10,ra10,ra10,ra10,ra10,ra10,ra10,ra11,ra11,ra11,ra11,ra11,ra11,ra11,ra11,ra11,ra11,r
```

```
a11,ra11,ra11,ra11,ra11,ra11,ra11,ra11,ra11,ra11,ra11,ra11,ra11,ra11,ra11,ra11,ra11,ra11,ra11,ra11,ra1
1,ra11,ra11,ra11,ra11,ra11,ra11,ra11,ra11,ra11,ra11]';
for year=1:N
transmrate(:,1)=HBVdata20171(:,5); % Age-specific transmission rate of HBV in 1992
transmrate(:,year+1)=(a1*exp(-a2*year)+(1-a1)).*transmrate(:,1);  % Method 2. For Method 1, set
a1=a2=0.
end
% Initial conditions
spop=HBVdata20171(:,6);
cpop=HBVdata20171(:,7);
rpop=HBVdata20171(:,8);
% Iterative procedure
for j=1:N
totalpop=sum(spop)+sum(cpop)+sum(rpop);
totalpopa=spop+cpop+rpop;
totalcpop=sum(cpop);
tadr(:,j)=HBVdata20172(:,j);  % Age-specific total death rate
nonhbvmr(:,j)=(totalpopa.*0.001.*tadr(:,j)-cpop.*hbvmr)./totalpopa; % Age-specific death rate of non-HBV
related diseases
survival=1-nonhbvmr;
womanhbvrate(j)=pirpy*0.8491*sum(cpop(16:50))/(sum(spop(16:50))+sum(cpop(16:50))+sum(rpop(16:50
)));
     snewpop(1)=birthrate(j)*totalpop*(1-vaccrate(j)-womanhbvrate(j));
     cnewpop(1)=birthrate(j)*totalpop*0.9*womanhbvrate(j);
     rnewpop(1)=birthrate(j)*totalpop*vaccrate(j)+birthrate(j)*totalpop*0.1*womanhbvrate(j);
    for i=2:maxage
     snewpop(i)=survival(i-1,j)*spop(i-1)-transmrate(i-1,j+1)*totalcpop*(spop(i-1))/(totalpop);
     cnewpop(i)=(survival(i-1,j)-clearancerate(i-1)-hbvmr(i-1))*cpop(i-1)+acap(i-1)*(transmrate(i-
1,j+1)*totalcpop*(spop(i-1)))/(totalpop);
     rnewpop(i)=survival(i-1,j)*rpop(i-1)+clearancerate(i-1)*cpop(i-1)+(1-acap(i-1))*(transmrate(i-
1,j+1)*totalcpop*spop(i-1))/(totalpop);
    end
    for i=1:maxage
     spop(i)=snewpop(i);
     cpop(i)=cnewpop(i);
     rpop(i)=rnewpop(i);
```

```
    ehbsagrate(i)=100*cpop(i)/(spop(i)+cpop(i)+rpop(i));
   end
hbsagrate(:,j)=ehbsagrate;
end
Ps=HBVdata20171(2:60,9); % Surveyed prevalence of HBsAg aged 1-59 years in 2006
Pm=hbsagrate(2:60,14); % Estimated prevalence of HBsAg by transmission model
Diff=Ps-Pm; % Objective function
end

%% Input parameter values and initial conditions
```

HBVdata20171.mat % Input parameter values and initial conditions

0.01809	0.3426	0	0.3	2.0199	16427937	1161507	3528870	NaN
0.0177	0.381	0	0.25	1.211	14106464	1719712	6108926	0.69
0.01712	0.3965	0	0.25	0.7501	17032866	2383780	6242989	1.01
0.01698	0.4519	0	0.25	0.4875	14990654	2377759	7687001	1.11
0.01657	0.5591	0	0.25	0.338	14906424	2648516	7693068	1.46
0.01564	0.5677	1.07E-05	0.06	0.2527	14033527	2717475	8598577	1.17
0.01464	0.6119	2.03E-05	0.06	0.2042	12094436	2256525	8013011	1.32
0.01403	0.7662	2.88E-05	0.06	0.1765	11073492	2066042	7336598	1.12
0.01338	0.8295	3.63E-05	0.06	0.1607	10990329	2050526	7281499	1.81
0.01286	0.8428	4.31E-05	0.06	0.1518	11456333	2137470	7590244	2.48
0.01241	0.8772	4.92E-05	0.06	0.1467	10473973	2483365	9077842	2.46
0.01229	0.9237	5.48E-05	0.06	0.1437	9214049	2184639	7985861	3.07
0.0124	0.9342	6.00E-05	0.06	0.1421	9126269	2163826	7909781	3.02
0.01209	0.984	6.49E-05	0.06	0.1411	9169940	2174180	7947632	3.5
0.0121	0.9842	6.98E-05	0.06	0.1406	8957988	2123927	7763931	5.57
0.01214	0.9843	7.47E-05	0.06	0.1403	8718734	2002325	8625077	7.21
0.01195	0.9847	7.97E-05	0.03	0.1401	9209646	2115067	9110714	7.21
0.0119	0.986	8.50E-05	0.03	0.14	9739907	2236846	9635281	7.21
0.01193	0.991	9.08E-05	0.03	0.14	10566184	2426606	10452681	7.21
0.0121	0.9967	9.71E-05	0.03	0.1399	11034686	2534201	10916149	7.21
0.01208	0.9967	0.000104	0.03	0.1399	10254005	2319199	11865144	8.17
0.01237	0.9967	0.000112	0.03	0.1399	10879434	2460656	12588843	8.17
NaN	NaN	0.000121	0.03	0.1399	10895815	2464361	12607797	8.17
NaN	NaN	0.000131	0.03	0.1399	11363961	2570243	13149499	8.17
NaN	NaN	0.000142	0.03	0.1399	10274841	2323912	11889253	8.17
NaN	NaN	0.000155	0.03	0.1399	8860942	2187522	11714516	8.25
NaN	NaN	0.000169	0.03	0.1399	9781020	2414664	12930895	8.25

NaN	NaN	0.000185	0.03	0.1399	9449523	2332827	12492641	8.25
NaN	NaN	0.000202	0.03	0.1399	9818691	2423964	12980697	8.25
NaN	NaN	0.000232	0.03	0.1399	10490542	2589826	13868911	8.25
NaN	NaN	0.000273	0.03	0.1399	5899954	1689789	8291730	7.95
NaN	NaN	0.000319	0.03	0.1399	4261186	1220434	5988626	7.95
NaN	NaN	0.000371	0.03	0.1399	5408291	1548973	7600752	7.95
NaN	NaN	0.000428	0.03	0.1399	5325434	1525242	7484307	7.95
NaN	NaN	0.000492	0.03	0.1399	7058068	2021481	9919332	7.95
NaN	NaN	0.000562	0.03	0.1399	6342507	1686211	10259908	8.25
NaN	NaN	0.000639	0.03	0.1399	6096871	1620907	9862558	8.25
NaN	NaN	0.000723	0.03	0.1399	6681641	1776373	10808505	8.25
NaN	NaN	0.000814	0.03	0.1399	6332570	1683569	10243832	8.25
NaN	NaN	0.001402	0.03	0.1399	5949648	1581766	9624401	8.25
NaN	NaN	0.001588	0.03	0.1399	5416796	1565249	9830511	8.63
NaN	NaN	0.00178	0.03	0.1399	4658552	1346145	8454434	8.63
NaN	NaN	0.001978	0.03	0.1399	4826015	1394535	8758348	8.63
NaN	NaN	0.002182	0.03	0.1399	4168460	1204527	7565005	8.63
NaN	NaN	0.002392	0.03	0.1399	3972716	1147965	7209767	8.63
NaN	NaN	0.002608	0.03	0.1399	3828596	1106319	6948211	8.06
NaN	NaN	0.002829	0.03	0.1399	3522188	1017779	6392136	8.06
NaN	NaN	0.003055	0.03	0.1399	3303178	954493.2	5994672	8.06
NaN	NaN	0.003286	0.03	0.1399	3179529	918763.4	5770273	8.06
NaN	NaN	0.003523	0.03	0.1399	3014767	871153.3	5471258	8.06
NaN	NaN	0.003765	0.03	0.1399	2804291	725266	6038596	8.61
NaN	NaN	0.004011	0.03	0.1399	2766865	715586.8	5958007	8.61
NaN	NaN	0.004262	0.03	0.1399	2613394	675894.8	5627530	8.61
NaN	NaN	0.004517	0.03	0.1399	2585222	668608.9	5566866	8.61
NaN	NaN	0.004777	0.03	0.1399	2665840	689459	5740466	8.61
NaN	NaN	0.005041	0.03	0.1399	2622723	678307.7	5647619	7.23
NaN	NaN	0.005308	0.03	0.1399	2618588	677238.2	5638714	7.23
NaN	NaN	0.00558	0.03	0.1399	2515379	650545.6	5416471	7.23
NaN	NaN	0.005856	0.03	0.1399	2525700	653214.8	5438695	7.23
NaN	NaN	0.006134	0.03	0.1399	2465762	637713.4	5309630	7.23
NaN	NaN	0.006417	0.03	0.1399	1910526	578568.1	5143730	NaN
NaN	NaN	0.006702	0.03	0.1399	1827273	560905.7	5011634	NaN
NaN	NaN	0.006991	0.03	0.1399	1820831	566559.5	5087009	NaN
NaN	NaN	0.007283	0.03	0.1399	1614294	509152.1	4593600	NaN
NaN	NaN	0.007577	0.03	0.1399	1606614	513652.4	4656150	NaN
NaN	NaN	0.007875	0.03	0.1399	1394519	451935.4	4115754	NaN
NaN	NaN	0.008174	0.03	0.1399	1316257	432403.4	3955869	NaN
NaN	NaN	0.008476	0.03	0.1399	1332105	443594.1	4076466	NaN

NaN	NaN	0.008781	0.03	0.1399	1207460	407589.8	3762123	NaN
NaN	NaN	0.009087	0.03	0.1399	1035212	354227	3283741	NaN
NaN	NaN	0.009395	0.03	0.1399	950360.2	343952	3243313	NaN
NaN	NaN	0.009705	0.03	0.1399	871342.3	319863.9	3028635	NaN
NaN	NaN	0.010017	0.03	0.1399	825938.6	307536.6	2923736	NaN
NaN	NaN	0.01033	0.03	0.1399	679046.3	256464.8	2447929	NaN
NaN	NaN	0.010644	0.03	0.1399	628110	240630.9	2305809	NaN
NaN	NaN	0.01096	0.03	0.1399	573233.6	222759.9	2142792	NaN
NaN	NaN	0.011277	0.03	0.1399	514854	202950.3	1959641	NaN
NaN	NaN	0.011594	0.03	0.1399	453153.1	181199.1	1756137	NaN
NaN	NaN	0.011912	0.03	0.1399	421131.3	170820.6	1661618	NaN
NaN	NaN	0.012231	0.03	0.1399	331514.9	136409.6	1331674	NaN
NaN	NaN	0.01255	0.03	0.1399	265432.3	116633.6	1156636	NaN
NaN	NaN	0.01287	0.03	0.1399	223588.8	99743.58	992546	NaN
NaN	NaN	0.013189	0.03	0.1399	200036.5	90598.09	904590.9	NaN
NaN	NaN	0.013509	0.03	0.1399	160058.9	73600.54	737324	NaN
NaN	NaN	0.013828	0.03	0.1399	128366.1	59931.33	602353.3	NaN
NaN	NaN	0.014147	0.03	0.1399	102699.2	48683.85	490884.2	NaN
NaN	NaN	0.014466	0.03	0.1399	85657.61	41230.18	417046	NaN
NaN	NaN	0.014784	0.03	0.1399	66057.68	32286.22	327595.6	NaN
NaN	NaN	0.015101	0.03	0.1399	49922.87	24777.08	252174.4	NaN
NaN	NaN	0.015417	0.03	0.1399	36840.24	18567.09	189541.1	NaN
NaN	NaN	0.015732	0.03	0.1399	25510.13	13910.08	144090.1	NaN
NaN	NaN	0.016046	0.03	0.1399	17202.77	9535.846	99064.1	NaN
NaN	NaN	0.016359	0.03	0.1399	10620	5984.834	62350.76	NaN
NaN	NaN	0.01667	0.03	0.1399	6057.936	3470.888	36261.26	NaN
NaN	NaN	0.016979	0.03	0.1399	4489.495	2615.347	27398.41	NaN
NaN	NaN	0.017287	0.03	0.1399	3037.582	1799.282	18900.36	NaN
NaN	NaN	0.017592	0.03	0.1399	2074.489	1249.534	13160.59	NaN
NaN	NaN	0.017896	0.03	0.1399	1502.182	920.1318	9716.628	NaN
NaN	NaN	0.018197	0.03	0.1399	979.1504	609.9523	6457.761	NaN
NaN	NaN	0.018496	0.03	0.1399	654.38	414.5904	4400.56	NaN
NaN	NaN	0.018792	0.03	0.1399	502.1592	323.5964	3443.326	NaN

HBVdata20172.mat % Age-specific total death rate (‰)

24.5024	24.5024	24.5024	34.08	34.08	34.08	34.08	34.08	34.08	34.08	16.02	16.02	16.02	16.02	16.02	4.6939	4.6939	4.6939	4.6939	4.6939	4.6939	4.6939
3.8612	3.8612	3.8612	3.19	3.19	3.19	3.19	3.19	3.19	3.19	1.67	1.67	1.67	1.67	1.67	1.4038	1.4038	1.4038	1.4038	1.4038	1.4038	1.4038
2.5104	2.5104	2.5104	2	2	2	2	2	2	2	1.23	1.23	1.23	1.23	1.23	0.8271	0.8271	0.8271	0.8271	0.8271	0.8271	0.8271
1.7478	1.7478	1.7478	1.43	1.43	1.43	1.43	1.43	1.43	1.43	0.69	0.69	0.69	0.69	0.69	0.5876	0.5876	0.5876	0.5876	0.5876	0.5876	0.5876
1.2554	1.2554	1.2554	1.05	1.05	1.05	1.05	1.05	1.05	1.05	0.63	0.63	0.63	0.63	0.63	0.4828	0.4828	0.4828	0.4828	0.4828	0.4828	0.4828

1.0307	1.0307	1.0307	0.85	0.85	0.85	0.85	0.85	0.85	0.85	0.64	0.64	0.64	0.64	0.64	0.4408	0.4408	0.4408	0.4408	0.4408	0.4408	0.4408
0.8525	0.8525	0.8525	0.69	0.69	0.69	0.69	0.69	0.69	0.69	0.54	0.54	0.54	0.54	0.54	0.4194	0.4194	0.4194	0.4194	0.4194	0.4194	0.4194
0.7377	0.7377	0.7377	0.62	0.62	0.62	0.62	0.62	0.62	0.62	0.34	0.34	0.34	0.34	0.34	0.3785	0.3785	0.3785	0.3785	0.3785	0.3785	0.3785
0.7175	0.7175	0.7175	0.58	0.58	0.58	0.58	0.58	0.58	0.58	0.45	0.45	0.45	0.45	0.45	0.3737	0.3737	0.3737	0.3737	0.3737	0.3737	0.3737
0.6392	0.6392	0.6392	0.5	0.5	0.5	0.5	0.5	0.5	0.5	0.54	0.54	0.54	0.54	0.54	0.3785	0.3785	0.3785	0.3785	0.3785	0.3785	0.3785
0.9037	0.9037	0.9037	0.5	0.5	0.5	0.5	0.5	0.5	0.5	0.5	0.5	0.5	0.5	0.5	0.4106	0.4106	0.4106	0.4106	0.4106	0.4106	0.4106
0.586	0.586	0.586	0.46	0.46	0.46	0.46	0.46	0.46	0.46	0.53	0.53	0.53	0.53	0.53	0.3843	0.3843	0.3843	0.3843	0.3843	0.3843	0.3843
0.5952	0.5952	0.5952	0.45	0.45	0.45	0.45	0.45	0.45	0.45	0.48	0.48	0.48	0.48	0.48	0.3911	0.3911	0.3911	0.3911	0.3911	0.3911	0.3911
0.6346	0.6346	0.6346	0.46	0.46	0.46	0.46	0.46	0.46	0.46	0.36	0.36	0.36	0.36	0.36	0.3873	0.3873	0.3873	0.3873	0.3873	0.3873	0.3873
0.6893	0.6893	0.6893	0.5	0.5	0.5	0.5	0.5	0.5	0.5	0.45	0.45	0.45	0.45	0.45	0.4036	0.4036	0.4036	0.4036	0.4036	0.4036	0.4036
0.7711	0.7711	0.7711	0.61	0.61	0.61	0.61	0.61	0.61	0.61	0.55	0.55	0.55	0.55	0.55	0.5108	0.5108	0.5108	0.5108	0.5108	0.5108	0.5108
0.9279	0.9279	0.9279	0.7	0.7	0.7	0.7	0.7	0.7	0.7	0.79	0.79	0.79	0.79	0.79	0.5724	0.5724	0.5724	0.5724	0.5724	0.5724	0.5724
1.0963	1.0963	1.0963	0.85	0.85	0.85	0.85	0.85	0.85	0.85	0.86	0.86	0.86	0.86	0.86	0.6385	0.6385	0.6385	0.6385	0.6385	0.6385	0.6385
1.2374	1.2374	1.2374	1.02	1.02	1.02	1.02	1.02	1.02	1.02	1.17	1.17	1.17	1.17	1.17	0.6627	0.6627	0.6627	0.6627	0.6627	0.6627	0.6627
1.3487	1.3487	1.3487	1.09	1.09	1.09	1.09	1.09	1.09	1.09	1.33	1.33	1.33	1.33	1.33	0.7136	0.7136	0.7136	0.7136	0.7136	0.7136	0.7136
1.4049	1.4049	1.4049	1.25	1.25	1.25	1.25	1.25	1.25	1.25	1.35	1.35	1.35	1.35	1.35	0.7775	0.7775	0.7775	0.7775	0.7775	0.7775	0.7775
1.4432	1.4432	1.4432	1.23	1.23	1.23	1.23	1.23	1.23	1.23	1.24	1.24	1.24	1.24	1.24	0.777	0.777	0.777	0.777	0.777	0.777	0.777
1.4401	1.4401	1.4401	1.28	1.28	1.28	1.28	1.28	1.28	1.28	1.47	1.47	1.47	1.47	1.47	0.7899	0.7899	0.7899	0.7899	0.7899	0.7899	0.7899
1.4023	1.4023	1.4023	1.29	1.29	1.29	1.29	1.29	1.29	1.29	1.18	1.18	1.18	1.18	1.18	0.8391	0.8391	0.8391	0.8391	0.8391	0.8391	0.8391
1.4675	1.4675	1.4675	1.36	1.36	1.36	1.36	1.36	1.36	1.36	1.26	1.26	1.26	1.26	1.26	0.8802	0.8802	0.8802	0.8802	0.8802	0.8802	0.8802
1.4488	1.4488	1.4488	1.4	1.4	1.4	1.4	1.4	1.4	1.4	1.55	1.55	1.55	1.55	1.55	0.9368	0.9368	0.9368	0.9368	0.9368	0.9368	0.9368
1.3161	1.3161	1.3161	1.36	1.36	1.36	1.36	1.36	1.36	1.36	1.26	1.26	1.26	1.26	1.26	0.9218	0.9218	0.9218	0.9218	0.9218	0.9218	0.9218
1.4529	1.4529	1.4529	1.42	1.42	1.42	1.42	1.42	1.42	1.42	1.37	1.37	1.37	1.37	1.37	0.9754	0.9754	0.9754	0.9754	0.9754	0.9754	0.9754
1.6509	1.6509	1.6509	1.41	1.41	1.41	1.41	1.41	1.41	1.41	1.45	1.45	1.45	1.45	1.45	1.0139	1.0139	1.0139	1.0139	1.0139	1.0139	1.0139
1.5072	1.5072	1.5072	1.48	1.48	1.48	1.48	1.48	1.48	1.48	1.73	1.73	1.73	1.73	1.73	1.1032	1.1032	1.1032	1.1032	1.1032	1.1032	1.1032
1.7031	1.7031	1.7031	1.57	1.57	1.57	1.57	1.57	1.57	1.57	1.56	1.56	1.56	1.56	1.56	1.1643	1.1643	1.1643	1.1643	1.1643	1.1643	1.1643
1.5847	1.5847	1.5847	1.56	1.56	1.56	1.56	1.56	1.56	1.56	1.78	1.78	1.78	1.78	1.78	1.2728	1.2728	1.2728	1.2728	1.2728	1.2728	1.2728
1.6839	1.6839	1.6839	1.64	1.64	1.64	1.64	1.64	1.64	1.64	1.89	1.89	1.89	1.89	1.89	1.2957	1.2957	1.2957	1.2957	1.2957	1.2957	1.2957
1.7755	1.7755	1.7755	1.61	1.61	1.61	1.61	1.61	1.61	1.61	1.82	1.82	1.82	1.82	1.82	1.3175	1.3175	1.3175	1.3175	1.3175	1.3175	1.3175
1.8183	1.8183	1.8183	1.74	1.74	1.74	1.74	1.74	1.74	1.74	1.94	1.94	1.94	1.94	1.94	1.5003	1.5003	1.5003	1.5003	1.5003	1.5003	1.5003
1.9853	1.9853	1.9853	1.92	1.92	1.92	1.92	1.92	1.92	1.92	1.9	1.9	1.9	1.9	1.9	1.6001	1.6001	1.6001	1.6001	1.6001	1.6001	1.6001
2.0654	2.0654	2.0654	1.89	1.89	1.89	1.89	1.89	1.89	1.89	2.02	2.02	2.02	2.02	2.02	1.6315	1.6315	1.6315	1.6315	1.6315	1.6315	1.6315
2.0276	2.0276	2.0276	2.06	2.06	2.06	2.06	2.06	2.06	2.06	2.09	2.09	2.09	2.09	2.09	1.7023	1.7023	1.7023	1.7023	1.7023	1.7023	1.7023
2.2901	2.2901	2.2901	2.13	2.13	2.13	2.13	2.13	2.13	2.13	2.2	2.2	2.2	2.2	2.2	1.7659	1.7659	1.7659	1.7659	1.7659	1.7659	1.7659
2.3715	2.3715	2.3715	2.3	2.3	2.3	2.3	2.3	2.3	2.3	2.26	2.26	2.26	2.26	2.26	1.945	1.945	1.945	1.945	1.945	1.945	1.945
2.7844	2.7844	2.7844	2.59	2.59	2.59	2.59	2.59	2.59	2.59	2.38	2.38	2.38	2.38	2.38	2.1553	2.1553	2.1553	2.1553	2.1553	2.1553	2.1553
2.8069	2.8069	2.8069	2.58	2.58	2.58	2.58	2.58	2.58	2.58	2.74	2.74	2.74	2.74	2.74	2.1761	2.1761	2.1761	2.1761	2.1761	2.1761	2.1761
2.9968	2.9968	2.9968	2.8	2.8	2.8	2.8	2.8	2.8	2.8	2.45	2.45	2.45	2.45	2.45	2.4744	2.4744	2.4744	2.4744	2.4744	2.4744	2.4744
2.2384	2.2384	2.2384	2.84	2.84	2.84	2.84	2.84	2.84	2.84	2.77	2.77	2.77	2.77	2.77	2.4923	2.4923	2.4923	2.4923	2.4923	2.4923	2.4923
3.6546	3.6546	3.6546	3.06	3.06	3.06	3.06	3.06	3.06	3.06	3.14	3.14	3.14	3.14	3.14	2.7618	2.7618	2.7618	2.7618	2.7618	2.7618	2.7618
3.8505	3.8505	3.8505	3.43	3.43	3.43	3.43	3.43	3.43	3.43	3.4	3.4	3.4	3.4	3.4	3.0683	3.0683	3.0683	3.0683	3.0683	3.0683	3.0683

4.2841	4.2841	4.2841	3.57	3.57	3.57	3.57	3.57	3.57	3.57	3.45	3.45	3.45	3.45	3.45	3.1311	3.1311	3.1311	3.1311	3.1311	3.1311	3.1311
4.4891	4.4891	4.4891	3.89	3.89	3.89	3.89	3.89	3.89	3.89	3.75	3.75	3.75	3.75	3.75	3.2856	3.2856	3.2856	3.2856	3.2856	3.2856	3.2856
5.1201	5.1201	5.1201	4.15	4.15	4.15	4.15	4.15	4.15	4.15	4.23	4.23	4.23	4.23	4.23	3.9356	3.9356	3.9356	3.9356	3.9356	3.9356	3.9356
5.7078	5.7078	5.7078	4.69	4.69	4.69	4.69	4.69	4.69	4.69	4.18	4.18	4.18	4.18	4.18	4.2444	4.2444	4.2444	4.2444	4.2444	4.2444	4.2444
6.3886	6.3886	6.3886	5.46	5.46	5.46	5.46	5.46	5.46	5.46	4.34	4.34	4.34	4.34	4.34	4.6796	4.6796	4.6796	4.6796	4.6796	4.6796	4.6796
6.6836	6.6836	6.6836	5.42	5.42	5.42	5.42	5.42	5.42	5.42	4.52	4.52	4.52	4.52	4.52	4.7254	4.7254	4.7254	4.7254	4.7254	4.7254	4.7254
7.219	7.219	7.219	6.16	6.16	6.16	6.16	6.16	6.16	6.16	5.28	5.28	5.28	5.28	5.28	4.9632	4.9632	4.9632	4.9632	4.9632	4.9632	4.9632
7.9314	7.9314	7.9314	6.54	6.54	6.54	6.54	6.54	6.54	6.54	5.22	5.22	5.22	5.22	5.22	5.4213	5.4213	5.4213	5.4213	5.4213	5.4213	5.4213
8.7945	8.7945	8.7945	7.4	7.4	7.4	7.4	7.4	7.4	7.4	6.57	6.57	6.57	6.57	6.57	6.1814	6.1814	6.1814	6.1814	6.1814	6.1814	6.1814
9.4472	9.4472	9.4472	8	8	8	8	8	8	8	6.47	6.47	6.47	6.47	6.47	6.3489	6.3489	6.3489	6.3489	6.3489	6.3489	6.3489
10.0189	10.0189	10.0189	8.38	8.38	8.38	8.38	8.38	8.38	8.38	7.51	7.51	7.51	7.51	7.51	6.8617	6.8617	6.8617	6.8617	6.8617	6.8617	6.8617
11.3274	11.3274	11.3274	9.59	9.59	9.59	9.59	9.59	9.59	9.59	7.49	7.49	7.49	7.49	7.49	7.2987	7.2987	7.2987	7.2987	7.2987	7.2987	7.2987
13.1331	13.1331	13.1331	10.42	10.42	10.42	10.42	10.42	10.42	10.42	9.02	9.02	9.02	9.02	9.02	8.1357	8.1357	8.1357	8.1357	8.1357	8.1357	8.1357
14.5667	14.5667	14.5667	12.09	12.09	12.09	12.09	12.09	12.09	12.09	9.9	9.9	9.9	9.9	9.9	9.2065	9.2065	9.2065	9.2065	9.2065	9.2065	9.2065
16.6788	16.6788	16.6788	14.57	14.57	14.57	14.57	14.57	14.57	14.57	11.29	11.29	11.29	11.29	11.29	10.322	10.322	10.322	10.322	10.322	10.322	10.322
17.0129	17.0129	17.0129	14.61	14.61	14.61	14.61	14.61	14.61	14.61	10.98	10.98	10.98	10.98	10.98	11.2087	11.2087	11.2087	11.2087	11.2087	11.2087	11.2087
19.3439	19.3439	19.3439	16.46	16.46	16.46	16.46	16.46	16.46	16.46	12.75	12.75	12.75	12.75	12.75	12.3292	12.3292	12.3292	12.3292	12.3292	12.3292	12.3292
21.4792	21.4792	21.4792	17.23	17.23	17.23	17.23	17.23	17.23	17.23	14.55	14.55	14.55	14.55	14.55	13.1738	13.1738	13.1738	13.1738	13.1738	13.1738	13.1738
22.8108	22.8108	22.8108	19.83	19.83	19.83	19.83	19.83	19.83	19.83	16.71	16.71	16.71	16.71	16.71	15.3854	15.3854	15.3854	15.3854	15.3854	15.3854	15.3854
24.81	24.81	24.81	22.13	22.13	22.13	22.13	22.13	22.13	22.13	18.17	18.17	18.17	18.17	18.17	16.6482	16.6482	16.6482	16.6482	16.6482	16.6482	16.6482
27.4563	27.4563	27.4563	22.69	22.69	22.69	22.69	22.69	22.69	22.69	18.86	18.86	18.86	18.86	18.86	17.2882	17.2882	17.2882	17.2882	17.2882	17.2882	17.2882
29.696	29.696	29.696	25.9	25.9	25.9	25.9	25.9	25.9	25.9	21.32	21.32	21.32	21.32	21.32	20.3639	20.3639	20.3639	20.3639	20.3639	20.3639	20.3639
34.9811	34.9811	34.9811	29.67	29.67	29.67	29.67	29.67	29.67	29.67	23.59	23.59	23.59	23.59	23.59	21.9164	21.9164	21.9164	21.9164	21.9164	21.9164	21.9164
37.7656	37.7656	37.7656	34.41	34.41	34.41	34.41	34.41	34.41	34.41	28.07	28.07	28.07	28.07	28.07	25.9505	25.9505	25.9505	25.9505	25.9505	25.9505	25.9505
45.0841	45.0841	45.0841	39.7	39.7	39.7	39.7	39.7	39.7	39.7	31.23	31.23	31.23	31.23	31.23	30.4337	30.4337	30.4337	30.4337	30.4337	30.4337	30.4337
45.9481	45.9481	45.9481	40.91	40.91	40.91	40.91	40.91	40.91	40.91	30.93	30.93	30.93	30.93	30.93	31.6532	31.6532	31.6532	31.6532	31.6532	31.6532	31.6532
50.1063	50.1063	50.1063	47.43	47.43	47.43	47.43	47.43	47.43	47.43	39.33	39.33	39.33	39.33	39.33	36.4968	36.4968	36.4968	36.4968	36.4968	36.4968	36.4968
53.2402	53.2402	53.2402	49.71	49.71	49.71	49.71	49.71	49.71	49.71	42.01	42.01	42.01	42.01	42.01	39.3136	39.3136	39.3136	39.3136	39.3136	39.3136	39.3136
60.0069	60.0069	60.0069	54	54	54	54	54	54	54	44.31	44.31	44.31	44.31	44.31	43.7193	43.7193	43.7193	43.7193	43.7193	43.7193	43.7193
61.7318	61.7318	61.7318	59.13	59.13	59.13	59.13	59.13	59.13	59.13	49.65	49.65	49.65	49.65	49.65	48.0828	48.0828	48.0828	48.0828	48.0828	48.0828	48.0828
69.324	69.324	69.324	64.49	64.49	64.49	64.49	64.49	64.49	64.49	54.22	54.22	54.22	54.22	54.22	48.1544	48.1544	48.1544	48.1544	48.1544	48.1544	48.1544
73.8933	73.8933	73.8933	69.71	69.71	69.71	69.71	69.71	69.71	69.71	57.81	57.81	57.81	57.81	57.81	58.1111	58.1111	58.1111	58.1111	58.1111	58.1111	58.1111
84.9363	84.9363	84.9363	79.54	79.54	79.54	79.54	79.54	79.54	79.54	67.8	67.8	67.8	67.8	67.8	63.5918	63.5918	63.5918	63.5918	63.5918	63.5918	63.5918
89.4673	89.4673	89.4673	92.38	92.38	92.38	92.38	92.38	92.38	92.38	75.27	75.27	75.27	75.27	75.27	70.543	70.543	70.543	70.543	70.543	70.543	70.543
104.5839	104.5839	104.5839	106.97	106.97	106.97	106.97	106.97	106.97	106.97	80.65	80.65	80.65	80.65	80.65	84.5218	84.5218	84.5218	84.5218	84.5218	84.5218	84.5218
107.215	107.215	107.215	110.7	110.7	110.7	110.7	110.7	110.7	110.7	86.87	86.87	86.87	86.87	86.87	87.5666	87.5666	87.5666	87.5666	87.5666	87.5666	87.5666
120.4086	120.4086	120.4086	122.13	122.13	122.13	122.13	122.13	122.13	122.13	96.99	96.99	96.99	96.99	96.99	96.4345	96.4345	96.4345	96.4345	96.4345	96.4345	96.4345
126.8699	126.8699	126.8699	131.07	131.07	131.07	131.07	131.07	131.07	131.07	107.57	107.57	107.57	107.57	107.57	103.9528	103.9528	103.9528	103.9528	103.9528	103.9528	103.9528
137.4924	137.4924	137.4924	141.94	141.94	141.94	141.94	141.94	141.94	141.94	116.79	116.79	116.79	116.79	116.79	114.9762	114.9762	114.9762	114.9762	114.9762	114.9762	114.9762
147.7601	147.7601	147.7601	151.5	151.5	151.5	151.5	151.5	151.5	151.5	136.66	136.66	136.66	136.66	136.66	122.0517	122.0517	122.0517	122.0517	122.0517	122.0517	122.0517
158.4262	158.4262	158.4262	161	161	161	161	161	161	161	127.37	127.37	127.37	127.37	127.37	130.2158	130.2158	130.2158	130.2158	130.2158	130.2158	130.2158

169.9384	169.9384	169.9384	176.98	176.98	176.98	176.98	176.98	176.98	176.98	155.15	155.15	155.15	155.15	155.15	142.0656	142.0656	142.0656	142.0656	142.0656	142.0656	142.0656
185.6209	185.6209	185.6209	193.68	193.68	193.68	193.68	193.68	193.68	193.68	141.98	141.98	141.98	141.98	141.98	157.0522	157.0522	157.0522	157.0522	157.0522	157.0522	157.0522
189.0077	189.0077	189.0077	209.37	209.37	209.37	209.37	209.37	209.37	209.37	167.35	167.35	167.35	167.35	167.35	171.6281	171.6281	171.6281	171.6281	171.6281	171.6281	171.6281
246.8808	246.8808	246.8808	236.51	236.51	236.51	236.51	236.51	236.51	236.51	208.3	208.3	208.3	208.3	208.3	191.442	191.442	191.442	191.442	191.442	191.442	191.442
246.8808	246.8808	246.8808	253.57	253.57	253.57	253.57	253.57	253.57	253.57	198.84	198.84	198.84	198.84	198.84	202.1502	202.1502	202.1502	202.1502	202.1502	202.1502	202.1502
246.8808	246.8808	246.8808	271.94	271.94	271.94	271.94	271.94	271.94	271.94	238.41	238.41	238.41	238.41	238.41	220.0383	220.0383	220.0383	220.0383	220.0383	220.0383	220.0383
246.8808	246.8808	246.8808	286.79	286.79	286.79	286.79	286.79	286.79	286.79	231.64	231.64	231.64	231.64	231.64	226.8529	226.8529	226.8529	226.8529	226.8529	226.8529	226.8529
246.8808	246.8808	246.8808	282.05	282.05	282.05	282.05	282.05	282.05	282.05	269.72	269.72	269.72	269.72	269.72	228.4619	228.4619	228.4619	228.4619	228.4619	228.4619	228.4619
246.8808	246.8808	246.8808	292.38	292.38	292.38	292.38	292.38	292.38	292.38	274.25	274.25	274.25	274.25	274.25	242.5241	242.5241	242.5241	242.5241	242.5241	242.5241	242.5241
246.8808	246.8808	246.8808	300.58	300.58	300.58	300.58	300.58	300.58	300.58	282.12	282.12	282.12	282.12	282.12	244.1339	244.1339	244.1339	244.1339	244.1339	244.1339	244.1339
246.8808	246.8808	246.8808	287.43	287.43	287.43	287.43	287.43	287.43	287.43	260.49	260.49	260.49	260.49	260.49	235.4337	235.4337	235.4337	235.4337	235.4337	235.4337	235.4337
246.8808	246.8808	246.8808	309.54	309.54	309.54	309.54	309.54	309.54	309.54	278.85	278.85	278.85	278.85	278.85	231.1997	231.1997	231.1997	231.1997	231.1997	231.1997	231.1997
246.8808	246.8808	246.8808	288.31	288.31	288.31	288.31	288.31	288.31	288.31	176.14	176.14	176.14	176.14	176.14	290.1999	290.1999	290.1999	290.1999	290.1999	290.1999	290.1999
246.8808	246.8808	246.8808	363.63	363.63	363.63	363.63	363.63	363.63	363.63	364.24	364.24	364.24	364.24	364.24	467.1693	467.1693	467.1693	467.1693	467.1693	467.1693	467.1693
